# The Scientific and Cultural Cost of Convenience Sampling in the Face of Rising Language Endangerment: Highlighting the Role of Language Acquisition

**DOI:** 10.1162/opmi_a_00199

**Published:** 2025-04-02

**Authors:** Sam Passmore, Birgit Hellwig, Rowena Garcia, Evan Kidd

**Affiliations:** Evolution of Cultural Diversity Initiative, The Australian National University; University of Cologne; Leibniz-Centre General Linguistics (ZAS); University of the Philippines Manila

**Keywords:** language endangerment, child language acquisition, linguistic diversity

## Abstract

We live in an unprecedented era of language endangerment and loss. In the midst of this crisis, it is becoming more and more evident that the psychological and cognitive sciences know very little about how most of the world’s languages are acquired, represented, and processed. Therefore, the opportunity to understand our most important and defining species-specific trait is being rapidly lost. In this Perspective, we highlight the extent of this problem, focusing on a key group at the heart of language transmission and loss—child language learners. We show that, due to sampling biases, very little is known about how children learn much of the vast corners of the linguistic design space, and that our opportunity to do so this is fast running out. We end by arguing for the greater integration of the academy, government, and community in addressing this problem.

## INTRODUCTION

Language is arguably the crowning cognitive evolutionary achievement of our species, allowing us to rachet up cognition to the point that humans occupy every environmental niche on Earth. Languages are also incredibly diverse (Evans & Levinson, 2009): currently numbering over 7,000 (Hammarström et al., 2024), they encode both the boundaries of the architecture of the human mind and the cultural and intellectual wealth of the communities that speak them (Hale et al., 1992). Yet, the behavioural and cognitive sciences have a somewhat blind tendency to rely on convenience sampling alongside the assumption that this small sample of the world’s population reflects invariant and thus normative perceptual, cognitive, and social processes (Arnett, 2009; Blasi et al., 2022; Henrich et al., 2010; Kidd & Garcia, 2022a; Nielsen et al., 2017; Singh et al. 2024; Thalmayer et al., 2021). Although there are increasingly louder calls to include broader linguistic diversity in cognitive studies (Blasi et al., 2022; Henrich et al., 2010; Kidd & Garcia, 2022a), languages are dying at an alarming rate (Bromham et al., 2022)—an emergency that has both scientific and human costs. Science is rapidly losing the ability to study many languages, and the riches they contain, but unarguably the greatest loss is felt by the communities whose languages are on the path to extinction (Evans, 2022).

The case for linguistic diversity in the study of human cognition is clear: an over-reliance on widely spoken and accessible languages like English skews our understanding of how humans perceive, think about, and organise their worlds (Blasi et al., 2022; Wierzbicka, 2014). Conversely, studying diverse languages reveals that while languages vary across a wide number of dimensions, such variation is not unbounded, presenting the opportunity to study both the cross-linguistic tendencies of cognition and the influence of language-specific structure on non-linguistic cognition (Levinson, 2003). Despite these points, the behavioural and cognitive sciences have been notably absent in combating the decline of linguistic diversity, except for a few interactions with the small subfield of language documentation (Hale et al., 1992; Himmelmann, 1998; McDonnell et al., 2018; Seifart et al., 2018).

We are at an important juncture in the history of our species where the behavioural and cognitive sciences can no longer afford to ignore language endangerment and can instead be part of the solution to stemming the flow of language loss. Crucial to this argument is the fact that a critical step in language endangerment is when a language transmission stagnates; that is, when it is no longer being acquired by children. Understanding the process of first language acquisition is critical to ensuring the longevity of a language and helping local communities maintain efforts to strengthen and revitalise their languages (Hellwig, 2022; Meek, 2019). This article is a call to those with an interest in the cognitive sciences and child-language acquisition to consider the urgent need to document, study, and strengthen vulnerable languages and to offer quantitative evidence for how their loss would limit our understanding of the diversity and mechanisms of human cognition and language development. Accordingly, we combine four data sets investigating: (i) language coverage in child language acquisition research (Kidd & Garcia, 2022a), the observed typological diversity (observed design space) of (ii) grammatical (Skirgård et al., 2023) and (iii) phonological systems (Moran & McCloy, 2019), and (iv) scales of language endangerment and loss (Bromham et al., 2022; Lewis & Simons, 2010). We first show that research on child-language acquisition focuses on large, unthreatened languages that are conveniently accessible to researchers in the Global North. We then demonstrate that this is a small slice of language diversity, paling in comparison to the vast corners of the design space of language that has not been studied, highlighting the deep cost involved in not studying diverse languages. Finally, we show that the speed at which languages are being studied is far outstripped by the rate at which transmission is stagnating and the rate at which languages are no longer spoken, demonstrating the urgency with which it is needed to collectively act. We end by arguing that nurturing the connections between science, government, and community is an important next step in empowering communities to maintain a multilingual future.

## THE CURSE OF CONVENIENCE

Kidd and Garcia (2022a) recently took stock of the language coverage in the field of child language acquisition. In a comprehensive analysis of journal articles published in four major child language journals from 1974–2020, they found only 103 languages represented, which constitutes only 1.5% of the world’s languages currently spoken today (103 divided by 7,667 observed languages; (Hammarström et al., 2024)^1^. They showed that in the sample, studied languages disproportionately belong to the Indo-European language family, are used in the Global North, and are typically the languages spoken by researchers and the public surrounding them. Thus, by and large, the majority of studies on language acquisition are based on convenience samples (see also: Doebel & Frank, 2024). Convenience sampling can be a useful tool for initiating a research program, but they are typically not representative of the population. Conclusions drawn from a convenience sample have limited generalisability and should not form the basis of a discipline (Doebel & Frank, 2024).

**Figure 1.**
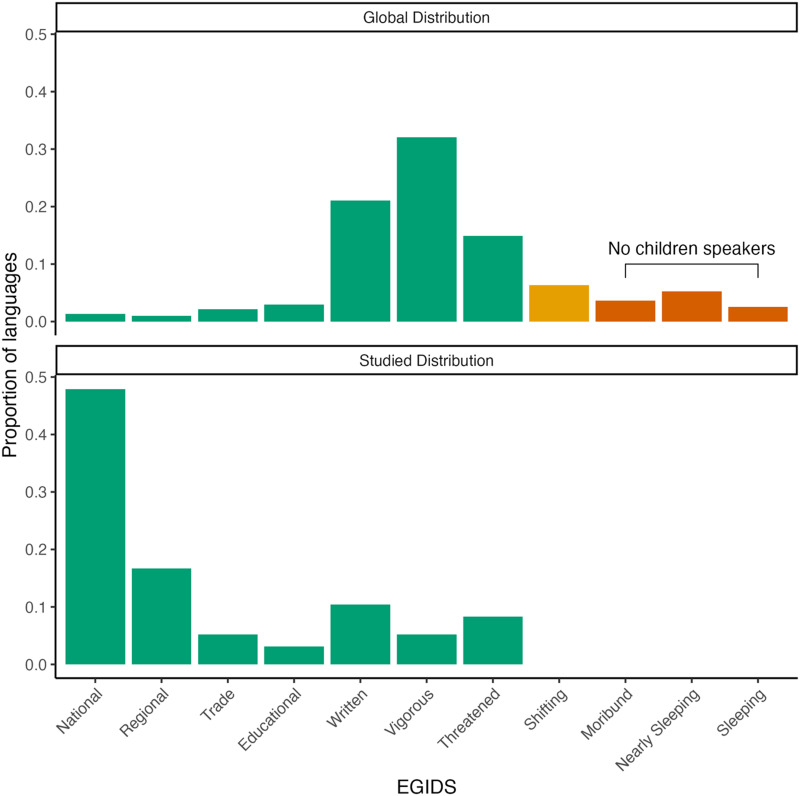
Top: Proportion of languages by endangerment category across all languages catalogued by Ethnologue (Eberhard et al., 2021). Bottom: Proportion of languages studied by endangerment category, from Kidd and Garcia (2022a). The EGIDS scale decreases in the level of linguistic vitality from left (National) to right (Sleeping), offering a measure of robustness to changes in the language’s environment. Green indicates languages where children are learning and speaking the language, orange is languages with some children speakers, and red is no children speakers.

The curse of convenience sampling takes effect when the underlying population is sufficiently different to the sample. We combined the collection of papers and languages by Kidd and Garcia (2022a) with the Expanded Graded Intergenerational Disruption Scale (EGIDS; Eberhard et al., 2021; Lewis & Simons, 2010) to show that 48% of studied languages are national languages (Figure 1). Children who learn national languages as their first language experience institutionalised and sustainable systems of language education (oral and literary), uninterrupted intergenerational language transmission, and a language that is compulsorily used across major forms of work, media, and government (Lewis & Simons, 2010). However, most languages are not national languages. The overwhelming majority of unstudied languages are categorised as written, vigorous, or threatened (87%). Children learning written languages experience informal and incipient literacy training, whereas children learning vigorous and threatened languages will rely solely on the oral or signed transmission of language. In the case of threatened languages, intergenerational transmission only occurs in some parts of the community. The differences in exposure and the transmission of languages to children between the typical studied language and the typical unstudied language, with the latter probably more representative of humans historically (Nettle, 1999), constitute differences between the sampled and underlying population. More generally, the ecological niches where languages are used differ across the scale, as minority languages are typically being used in fewer settings overall and alongside other languages in contexts of high multilingualism. The difference in the social environments means that, at least on this dimension, conclusions from the majority of child language acquisition research rely on a sample that is different to the global distribution of languages, making generalisation to all humans difficult. Worse still, it leaves the languages most vulnerable to endangerment as unstudied.

## EXISTING LITERATURE COVERS A FRAGMENT OF THE DESIGN SPACE OF LANGUAGE

The tendency to rely on convenience samples is not specific to child language research, but all research in the cognitive sciences (Blasi et al., 2022; Majid & Levinson, 2010). In many cases, researching convenient languages is an efficient use of scarce academic resources, but the biased distribution of resources to the Global North produces a biased study sample. Here we probe the structural variance of those languages studied, aiming to investigate how language coverage in child language research maps onto estimates of structural diversity in the world’s languages.

The totality of linguistic diversity has previously been conceptualised as an ontological ‘design space’ (Dennett, 1995; Evans, 2013). A design space for linguistics would contain all possible organisations of language, or linguistic phenomena, and has been a driving force behind theoretical advancement in general, exemplified in phonology by the development of the International Phonetic Alphabet (IPA) (Evans, 2013). Ideally, the design space would be built from theoretical foundations (e.g., Nerlove & Romney, 1967), but as in biology (Pigot et al., 2020), approximations of a linguistic design space have been derived from observed diversity via the development of large comparative databases (e.g., Passmore et al., 2021; Skirgård et al., 2023). When triangulated with the language coverage in child language acquisition research and language endangerment data, these databases allow us to quantify the scope of typological coverage in the field by identifying understudied and untouched parts of the design space.

**Figure 2.**
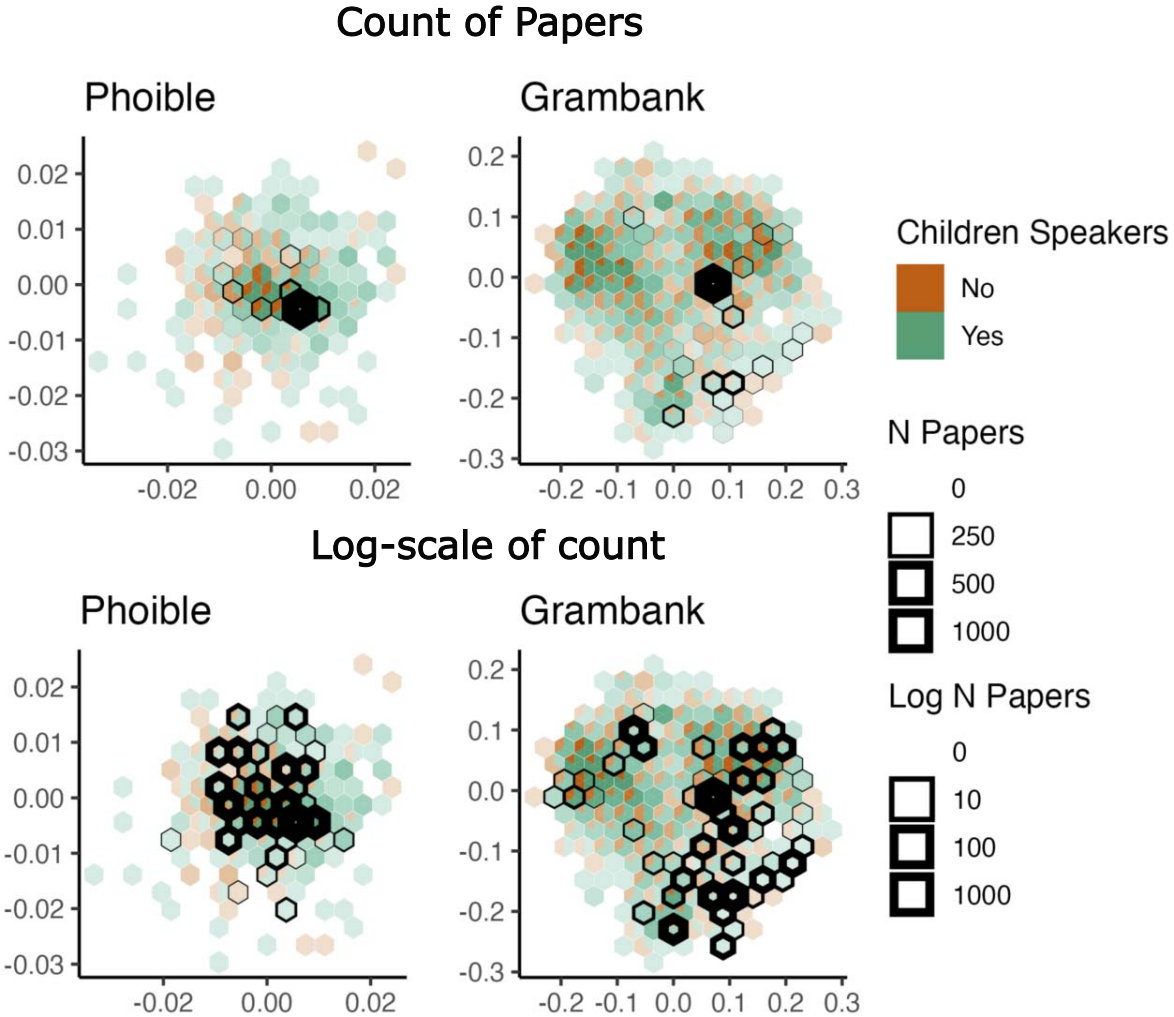
Hexagonal Bin (Hexbin) plots of phonetic and grammatical diversity, observed from languages in the Phoible and Grambank datasets, respectively. Each Hexbin shows the proportion of languages within a bin that have child speakers (and is therefore accessible to child language acquisition studies), and are shaded by the number of languages within a cell (darker corresponds to more languages). Cells that contain languages with at least one paper written about phonology or grammar, respectively, are highlighted in black. In the top row, linewidth indicates the raw count of papers published on this grammatical type, and in the bottom row, linewidth shows counts on a log scale. The raw scale shows how extreme the publication focus is on a particular typological structure, whereas the log scale more clearly shows the thin breadth of linguistic diversity studied, and the extent of completely unstudied diversity in each domain.

Figure 2 maps the language coverage in child language acquisition research using the data from Kidd and Garcia (2022a) onto the phonological and grammatical design space using the Phoible (Moran & McCloy, 2019) and Grambank (Skirgård et al., 2023) databases. We chose these two domains because they are bounded formal systems, enabling the generative capacity unique to language (Hockett, 1960). Their boundedness means that any one language only uses a subset of possible grammatical features or phonemes, but when aggregated across many languages we can make practical estimates of the design space of language. Phoible contains the cross-linguistic phonological inventory of 2,186 languages, from 176 language families, and a total of 3,183 segment types (Moran & McCloy, 2019). Grambank contains the grammatical structure of 2,467 languages, from 215 language families, across 195 features (Skirgård et al., 2023). We built observed design spaces of phonological and grammatical diversity and cross-referenced them with data on language endangerment and whether the languages and topic (i.e., phonology or grammar) were reported in Kidd and Garcia (2022a).

To visualise the design space, we downloaded the Phoible and Grambank datasets via their open-access repositories (Phoible: https://zenodo.org/records/2626687; Grambank: https://zenodo.org/records/7844558). The Phoible dataset was processed as follows: (i) We reduced the Phoible dataset to one phonological inventory per language (i.e., one doculect per Glottocode), with inventories chosen at random. (ii) We identified all phonemes used in the dataset and showed whether they are present (1) or absent (0) in each language, for a total of 1,343 possible phonemes. The Grambank data was processed following steps laid out in the Material and Methods of Skirgård et al. (2023). The key steps are: (i) limiting data to one dialect per language, leaving 2,430 languages (keeping the dialect with the most complete data), (ii) making dummy variables from the six non-binary variables, bringing the total to 201 features, and (iii) using random forests to impute the missing data, which totals around 24% of the dataset. This means each dataset is structured so that rows are languages and columns are either a grammatical feature or a particular phoneme, where 1 indicates the presence and 0 is the absence of a feature. All code and data used in this paper is available at: doi.org/10.5281/zenodo.14498105.

To project the datasets into a 2-dimensional space, we calculated the Gower distance between all languages (Gower, 1971). For a set of binary variables, this is the sum of features in common, divided by the total number of features. Each dataset is projected into a 2-dimensional space using classical multidimensional scaling (MDS). MDS translates information from a pairwise distance matrix into an abstract 2-dimensional space, where the proximity of points is indicative of similarity. Using the cartesian points from MDS, the space is divided into hexagonal bins (from here, Hexbins). Each Hexbin can be conceptualised as a collection of similar phonological inventories or grammars. Bin sizes were chosen so that the average number of languages in each bin is approximately 5. It should be noted that the size of Hexbins influences the display and interpretation of the plots; however, the total number of bins and the number of studied bins show a strong correlation (for more detail, see the supplementary materials). We added two pieces of metadata to the languages in the Grambank and Phoible datasets: whether that topic (i.e., phonology or grammar) has been studied for a given language in the top four child language acquisition journals or not (Kidd & Garcia, 2022a), and whether a language is currently being spoken by children or not (as taken from EGIDS). For each Hexbin, we counted the number of papers that have been published on languages in that part of phonological and grammatical space, and we calculated the proportion of languages in that bin with and without child-aged speakers (defined as ‘Shifting’ or above on the EGIDS scale). All languages from Kidd and Garcia (2022a) and EGIDS were matched to Phoible and Grambank using Glottocodes.

We have collectively studied 26% of the phonological cells, and 27% of the grammatical cells, but cumulative research effort is biased towards a few corners of the design space. As Kidd and Garcia (2022a) observed, the data coverage is skewed towards English and closely related languages. As a result, 51% of all papers appear in one cell of the observed phonological space, and 40% of all papers appear in one cell of the observed grammatical space. Of the cells that have been studied, the majority have less than ten papers written about them (Phonological: 17 cells out of 28 studied (61%); Grammatical: 27 of 49 (55%)). If it is acceptable to take these data as representative of the field in general, it is clear that research on child language acquisition is investing heavily in a small area of the linguistic design space, which has consequences for the generalisability of theories on language development.

There are two important things to note. Firstly, it is very likely that Figure 2 *underestimates* the diversity in the design space of language. The models are limited to the languages recorded and the parameterisation of the Phoible and Grambank data sets. Both data sets only cover around one-third of global languages. Additionally, Grambank contains a proportion of imputed data, which causes a slight regression to the mean. Although there is likely some redundancy in undocumented languages, in that they may contain features shared with languages already in the dataset, in all likelihood less is known about acquisition across the entirety of the linguistic design space than is estimated here. This starker picture could be offset by studies on languages not published in the journals studied by Kidd and Garcia (2022a), especially since their analysis was limited to English language publications. However, on best estimates, a more exhaustive search would still fall far short of even adequate coverage (Kidd & Garcia, 2022b). Secondly, at least for grammar, the existing literature has been repeatedly studying a part of the design space that is also unrepresentative of the world’s languages. In an analysis of the *World Atlas of Language Structures*, Cysouw (2011) reported that the languages of northwestern Europe (e.g., English, Dutch, German, French), which feature heavily in our dataset, have an exceptional number of rare structural features when compared to other languages of the world.

Although existing research only covers a small portion of the observed design space, we are not stating that languages inhabiting the same area of design space as a studied language are less valuable. On the contrary, comparisons of closely related languages can yield important insights because they hold many variables constant (e.g., culture, many linguistic variables), allowing researchers to test specific hypotheses concerning how variation affects acquisition (see Christiansen et al., 2022). Similarly, work on well-studied languages can also be valuable for the precise reason that there is a lot of information about them (e.g., how individual acquisition trajectories vary; Kidd & Donnelly, 2020).

## TIME IS RUNNING OUT

It is already the case that some parts of the design space are inaccessible to child language acquisition research. Of the 109 bins in the Phoible plot and 184 in the Grambank plot, 16 phonological bins (15%), and 9 grammatical bins (5%) contain only languages with no children speakers. In a further 17 phonological cells and 45 further grammatical cells, half or fewer of the languages are still being acquired by children. Bromham et al. (2022) recently reported on predictors of language endangerment. Assuming no action is taken to mitigate language loss, they showed that language loss could triple within the next 40 years, with at least one language lost per month—a total loss of 1,500 languages. They further show that an average of 72 languages per year will move from having some children speakers to no children speakers. Therefore, the opportunity to study languages and the riches they contain is being rapidly lost. Worse still, the opportunities to stem the flow of language loss by understanding how they are transmitted across generations and therefore acquired by children are passing by at an alarming rate.

**Figure 3.**
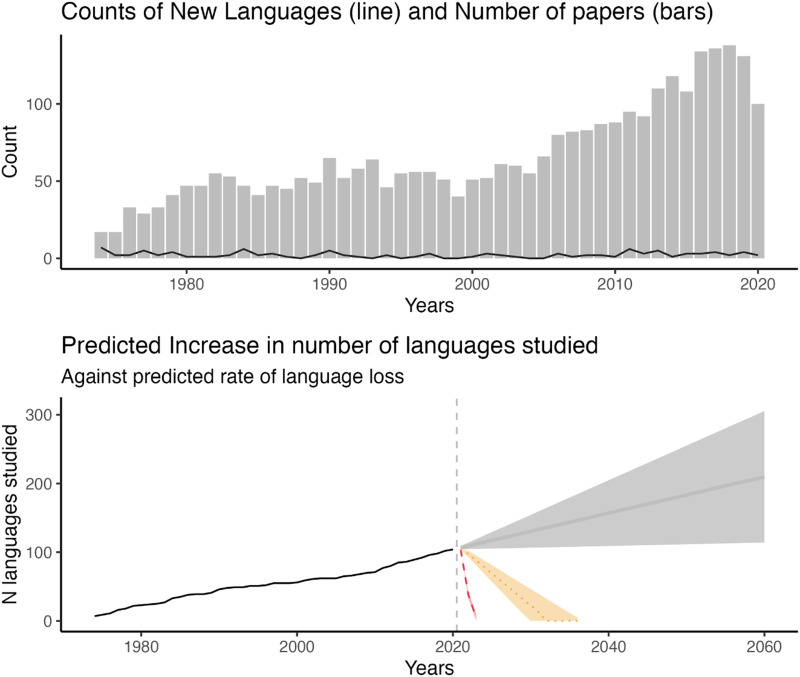
Top: A bar plot showing the number of papers published per year in the top four child language acquisition journals. The overlaid line shows the number of languages that had not previously been published in these journals per year. Bottom: Up to 2020, the graph shows the cumulative number of languages being studied per year, beyond 2020 the graph shows ARIMA model predictions (grey) on the number of new languages being studied per year. The graph also shows the net number of languages studied, accounting for the rate of language loss (yellow) and the rate of languages stagnating (red).

We show this by juxtaposing the findings of Kidd and Garcia (2022a) against data from Bromham et al. (2022). Between 1974 and 2020, the number of papers published across the four journals of interest has increased from an average of 51 papers per year (1980 to 2000; range: 40–65) to an average of 90 papers per year (2001 to 2020; range: 52–138). Since 2013, over 100 papers have been published per year, showing that the field of child language acquisition has grown considerably over the past five decades. Despite the increase in research productivity, child language acquisition journals have consistently only published two unstudied languages per year since 1974. Using an ARIMA model (Autoregressive Integrated Moving Average model), we found that there is no statistical relationship between the number of new languages studied per year, and the number of papers published (*β* = 0.006, *p* = 0.4; Figure 3). Thus, increased research effort has not led to studying greater linguistic diversity. This is somewhat of a wasted opportunity with a high price: in the bottom half of Figure 3 we used the Kidd and Garcia (2022a) data to determine the rate at which the acquisition of previously unstudied languages appears in the literature, juxtaposing it against Bromham et al.’s (2022) predicted rates of language stagnation and loss. It shows that the rate at which languages stagnate is far outstripping the rate at which researchers are investigating previously understudied languages. Time is running out.

## WHAT CAN BE DONE?

The triangulation of linguistic diversity in child language acquisition research against phonological diversity, grammatical diversity, and language endangerment shows that the sample of languages that form the basis of our understanding of child language acquisition research is small and unrepresentative of global language diversity. Many languages that have not been studied are healthy and have the potential to contribute new insights. However, the current rate of language endangerment means the ability to study the acquisition of a significant proportion of languages is being lost. Poor sampling and biased resource distribution reduces our capacity to build fully representative theories of children’s privileged capacity for language (Pye, 2021), and once these languages are lost to child language they are lost to the broader enterprise of the cognitive science of language.

This scientific problem is overshadowed by the loss of cultural knowledge and connection that language loss entails (e.g., Cámara-Leret & Bascompte, 2021; Kik et al., 2021). In many parts of the world, insider linguists (i.e., community members), language activists, and Indigenous language committees are developing community-led responses to this threat, and their voices are increasingly being heard within academia; for instance, they are being published in peer-reviewed journals such as *Language Documentation and Conservation*. Building on earlier discussions about how academic outputs may benefit communities, the field is increasingly moving towards co-designing projects and developing models for participatory research that incorporate Indigenous views into the research aims and design from the beginning. In this respect, language transmission emerges as a key variable in maintaining linguistic diversity, and several community initiatives such as the highly successful language nests programs and master/apprentice programs directly target this issue (Hinton, 2001; for further initiatives, cf. Child Language Research and Revitalization Working Group, 2017; Grenoble & Whaley, 2006; Lee & Van Way, 2016; O’Grady & Hattori, 2016).

If children continue to acquire a language, that language will survive for at least another generation (McDermott et al., 2024). Creating societies in which minority languages are valued by connecting people to their family and identity will ensure their survival for even longer (Meek, 2019). Child language acquisition research is one of the few research domains that deals directly with the process of language transmission, begging the question: is there an equitable framework in which science can better work with communities to help them maintain their languages?

What is clear from recent discussions is that the cognitive and psychological sciences have a diversity problem (Arnett, 2009; Blasi et al., 2022; Medin et al., 2017; Thalmayer et al., 2021, Moriguchi, 2022; Singh et al., 2024). The producers of research are mostly in the Global North and mostly study members of their own communities. The study of language acquisition is no different (see Kidd & Garcia, 2022a), and unless there is significant disruption to the status quo, this pattern is unlikely to change. It is currently the beginning of the United Nations’ *International Decade of Indigenous Languages*^2^ (2022–2032), and so there is, at face value, broad consensus that strengthening vulnerable languages is an important humanitarian concern (United Nations, 2016). Figure 2 suggests that an important step-change is required in capacity to increase the number of languages for which there is knowledge of acquisition, alongside methods that allow tractable and timely collection of data. An example of the step-change is the Acquisition Sketch Project (Hellwig et al., 2023). The Acquisition Sketch Project is an initiative that integrates perspectives from language documentation, socialisation and acquisition into a model that facilitates small-scale acquisition studies in close cooperation with local communities. To this effect, it is in the process of establishing a support infrastructure and a worldwide network of collaborators encompassing junior and senior academics from across the Global North and South (see also Pye, 2021). We agree that the best way forward is to build on the experiences already gained in the last three decades within the field of language documentation and to substantially expand their approach. We suggest two practical changes that are needed to achieve this. The first is an increase in the number of researchers working on languages. The best way to achieve this goal is to build strong partnerships with communities, and to conceive of researcher training as a two-way process, incorporating Indigenous perspectives into research designs, aims, and methodologies as well as offering scientific training for insider linguists as has been implemented in some parts of the world for language documentation (Lachler & Rice, 2013, see Box 1), in addition to embedding documentation within well-supported educational contexts (Disbray et al., 2022). The second is to increase efforts in building partnerships with governments and non-government organisations (NGOs), who could be lobbied to provide ongoing and sustainable funding for documentation programs—which often go unfunded because of unfairly used academic metrics (Rafiq et al., 2024).

**Figure 4.**
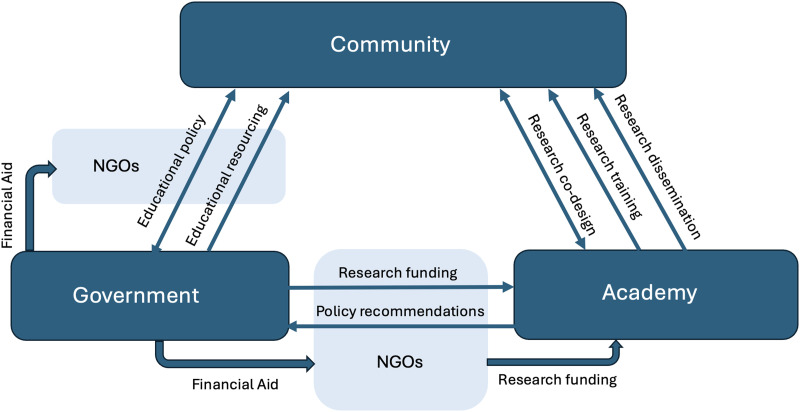
**Ecology of connections between key stakeholders in addressing language diversity in child language and its role in language maintenance.** Unidirectional arrows indicate that one stakeholder plays a primary role in providing resources or knowledge to another stakeholder. Bidirectional arrows indicate that stakeholders have equal hands in iterative co-design or policy and research. NGOs play important roles in lobbying the government and liaising between the government and academia and language communities, especially in the Global South, where governments lack funds to adequately resource education and research. We use the box “Academy” to include both the Global North and Global South, but the inequalities in resource allocation severely limit Global South academics’ ability to act (Aravena-Bravo et al., 2024). A more detailed model will have to represent the internal diversity within academia. Addressing the inequality would include furthering structures that facilitate North-South collaboration and increasing the visibility of research originating in the Global South (such as recognition of publications in languages other than English; see Kidd & Garcia, 2022b; Singh et al., 2023, 2024 for proposed steps towards achieving a fairer and more global science). Our model does not explicitly integrate this aspect but allows for this within the “Academy” box.

Box 1. Community- and Researcher-driven language programs. Studying endangered and unstudied languages is a task best accomplished through the combined efforts of researchers, communities and funding bodies (Figure 4). The most difficult hurdle is starting and maintaining a collaboration at eye level, and not losing sight of either scientific or community goals. While outsider researchers can go some way to document the language, community buy-in and collaboration are essential to the successful documentation, maintenance, or revitalisation of a language (indeed, it is the community’s choice as to whether they want to continue to speak their language). At the same time, responding to community needs and promoting communities’ capacities to study their own language also serves to diversify the discipline, which is currently dealing with its colonial past (Charity Hudley et al., 2020; Woods, 2022). Community-driven linguistic training programs have been successfully implemented in some parts of the world for language documentation (Lachler & Rice, 2013).

Local initiatives, such as the *Kulu Language Institute* (https://kululanguage.com), have been launched by communities to address identified concerns and help establish positive attitudes towards vernacular languages. Implementing such a program, however, is an enormous task that involves gaining access to funds; identifying, recruiting, training and retaining volunteers and staff; developing resources and infrastructure; and creating, safeguarding and disseminating results (McDougall & Zobule, 2021). Collaborative examples, such as *CoLang* (Institute on Collaborative Language Research, www.colanginstitute.org) and the *auto-documentation des langues amazighes* project (Mettouchi, 2021), allow academic linguists to aid communities in these tasks and facilitate documentation through linguistic training, introducing new technologies, and enabling the appropriate governance of new materials.

Equally, community-based linguistic initiatives are partners in scientific research, providing a frame where cooperation between communities and outsider researchers can flourish. Ameka (2006) details the importance of both local and outside engagement in language description, whose arguments can be equally applied to child language acquisition. All researchers will have some bias they carry—outsider researchers may fixate on points of difference rather than points of importance, insider researchers may gloss over familiar details, and communities will likely lack the expertise required to examine the development of child language. If successful, this cooperation will not only address the sampling problem in our disciplines, but can lay the foundations for community-driven language maintenance efforts (e.g., via educational materials and programs), and in some cases may serve as a form of restorative linguistic justice.

Figure 4 emphasises an interconnected solution to studying and maintaining the transmission of languages in contexts of potential language endangerment. No doubt tackling this problem effectively is an enormous task. However, to not attempt to act would be to ignore the inequities that exist within the geopolitics of language loss. Languages diversify unevenly across the globe, with greater diversity in the tropics (Gorenflo et al., 2012), within the Global South. A key finding from Bromham et al. (2022) is that language loss is significantly linked to modernisation: access to education and road density (indexing ease of population movement) drives language loss and not, as typically assumed, the presence of other languages. Thus, just as industrialisation has influenced biodiversity via habitat destruction and climate change, it threatens linguistic diversity, and minority peoples within the Global South are disproportionately affected.

Unlike biodiversity, however, preserving the conditions of the past is neither a viable nor a desirable solution to language endangerment (involving child language or otherwise) (McDermott et al., 2024). Communities that speak endangered languages are likely to face many economic, environmental, social, and health challenges, which makes adopting more widely spoken languages attractive. Governments in the Global South face the challenge of fostering the participation of diverse linguistic groups in their country’s administrative, economic and political system within an ecosystem that relies on vehicular languages while attending to priorities such as healthcare, agriculture, and development. Despite external pressures, a community’s ability to connect to its linguistic and cultural heritage is implicated in its members’ well-being and success within the educational system (Alkateb-Chami, 2024; Whalen et al., 2016). Thus, excluding communities from research also excludes them from maximally benefitting from that research, such as the co-creation of educational resources that contribute to the community, violating the Principle of Justice, a recognized principle within research ethics (Beauchamp & Childress, 2001). So, for those outside the communities, we must acknowledge the agency of a community in strengthening their language. A promising strategy is to empower communities to promote new cultural or linguistic environments that encourage linguistic diversity, where Indigenous languages find their place alongside languages of wider communication. We suggest this is where governments and NGOs in the Global North would ideally contribute, providing funding for community infrastructure, research projects and training. This obligation is perhaps most obvious in contexts of colonisation and occupation, where the loss of Indigenous languages is great (e.g., in Australia and the United States, see Skirgård et al., 2023), and large sums of money has funded English teaching programs, which has been criticised as linguistic imperialism (Pennycook, 1993; Piller, 2016) and a direct threat to linguistic diversity (Skutnabb-Kangas, 2003).

Another source of funding comes from philanthropic donations. NGOs such as the *Endangered Language Fund* (https://www.endangeredlanguagefund.org) and the *Endangered Languages Documentation Programme* (https://www.eldp.net) have successfully secured donations and have been able to support hundreds of projects supporting the documentation of endangered languages. To date, however, studies of child language have mostly been outside of their remit, largely because highly endangered languages (i.e., those without child speakers) have been seen rightly as a priority.

## CONCLUSION

In this Perspective piece we have considered the consequences of sampling biases in the field of child language acquisition. Addressing the sampling problem will improve scientific generalisability. More importantly, diversifying child language acquisition research will be an important step in addressing further language loss. Broadening the scope of child language acquisition research, and the diversity of researchers, can contribute to efforts to document, strengthen, and revitalize these languages, ensuring that linguistic diversity remains a vibrant aspect of our global heritage, while also providing deeper insights into the scientific question of how children master language.

## ACKNOWLEDGMENTS

We would like to thank Amina Mettouchi, Alphaeus Zouble, Guy Lavender-Forsyth, and two anonymous reviewers for their comments on the manuscript.

## AUTHOR CONTRIBUTIONS

S.P.: Conceptualization; Formal analysis; Writing – original draft; Writing – review & editing. B.H.: Writing – original draft; Writing – review & editing. R.G.: Investigation; Writing – original draft; Writing – review & editing. E.K.: Conceptualization; Investigation; Writing – original draft; Writing – review & editing.

## DATA AVAILABILITY STATEMENT

The data and analysis that support the findings of this study are openly available on Zenodo (doi.org/10.5281/zenodo.14498105).

## Notes

^1^ Kidd and Garcia (2022a) only provide us with a sample of the child language acquisition literature—covering four English journals and 3,123 articles published between 1974 and 2020. Papers on lesser-studied languages are known to exist outside these journals (e.g., Meakins & Algy, 2016; Pye, 2013), and the journals examined may have systematic biases (Arunachalam et al., 2022; Paradis, 2022; Singh, 2022). These limitations mean that the numbers from Kidd and Garcia (2022a) are an underestimate of studied diversity. Reviews of language acquisition research within Papuan languages (860 languages) found research on eight languages, of which six were outside the scope of Kidd and Garcia (2022a) and Hellwig et al. (in press). This review highlights both the possibility of uncaptured data in the Kidd and Garcia dataset, but also the sparsity of child-language acquisition research in general. Despite these limitations, Kidd and Garcia (2022a) offer the broadest snapshot of linguistic diversity in child language acquisition currently available.^2^ https://www.un.org/development/desa/indigenouspeoples/indigenous-languages.html.

## Supplementary Material


