# Supplementary material for: The Scientific and Cultural Cost of Convenience Sampling in the Face of Rising Language Endangerment: Highlighting the Role of Language Acquisition
Source: Open Mind (Camb). 2025 Apr 2;9:501–14. doi: 10.1162/opmi_a_00199 (PMC11984793; doi:10.1162/opmi_a_00199)
Supplement: Supplementary file 1 [file opmi-09-501-s001.pdf]

Supplementary Material: The scientific and cultural cost of convenience sampling in the face of rising language endangerment: Highlighting the role of language acquisition

Sam Passmore, Birgit Hellwig, Rowena Garcia, and Evan Kidd

This document details the data, code, figures, and decisions used in Passmore, Hellwig, Garcia, and Kidd (2025).

Packages necessary for this report are listed in the next code chunk. They will require installation if not already installed. Details for installing rcldf are in the comment below, but all other packages can be installed using `install.packages()`.

```
library(dplyr)
library(tidyr)
library(ggplot2)
library(ggpubr)
library(patchwork)
library(forecast)
library(assertthat)

# To install rcldf:
# devtools::install_github("SimonGreenhill/rcldf", dependencies = TRUE)
library(rcldf)

# To Install ggpubfigs
# devtools::install_github("JLSteenwyk/ggpubfigs")
library(ggpubfigs)

## Parameters
font_size = 12
```

Data Sources

There are four key sources of data used in this project:

- Grambank (Skirgård et al. 2023)
- Phoible (Moran and McCloy 2019)
- Paper descriptions from the top four Child Language Acquisition Journals (Kidd and Garcia 2022)
- The Expanded Graded Intergenerational Disruption Scale (EGIDS) (Lewis and Simons 2010; Bromham et al. 2021)

Grambank, Phoible, and Glottolog are included as submodules as part of the repository, linking directly to their GitHub repositories. We include the data from Evan & Garcia (2022) as a csv file in this repository, but is also available from the original source: https://osf.io/jmxnw

Grambank

Grambank contains the grammatical structure of 2,467 languages, from 215 language families, across 195 features ranging from word order to verbal tense, to nominal plurals (Skirgård et al. 2023).

For the analysis, we used the GitHub repository created for the Grambank release paper (https://github.com/grambank/grambank-analysed), which contains some convenience scripts that we can use in the repository to make our analyses easier. In the Grambank release paper (Skirgård et al. 2023), there is a series of data curation steps that are used to create a dataset ready for analysis.

The key steps are:

1) Limiting data to one dialect per language, leaving 2,430 languages and keeping the dialect with the most complete data.

2) Converting the six non-binary variables to binary variables, bringing the total to 201 features.

3) Using random forests to impute the missing data, which totals around 24% of the dataset.

4) Reducing the dataset to its most complete state (1,509 languages, and 114 features).

These steps are performed within the system command in the text below, and produce a datafile where the rows are languages and the columns are Grambank features, which we will use for the multi-dimensional scaling analysis later.. More details can be found in the Grambank release paper, or within the Github repository.

```
# Create the file if it doesn't exist (takes a minute or so)
gb_dir = "submodule/grambank-analysed/R_grambank"
if(!file.exists(
  paste0(gb_dir, "/output/GB_wide/GB_wide_imputed_binarized.tsv")
  )){
  system(
  "cd ./submodule/grambank-analysed/R_grambank/;
  git submodule update --init
  mkdir -p output/non_GB_datasets
    mkdir -p output/coverage_plots
    mkdir -p output/GB_wide
    Rscript make_glottolog-cldf_table.R
    Rscript unusualness/processing/assigning_AUTOTYP_areas.R
    Rscript make_wide.R
    Rscript make_wide_binarized.R
  Rscript impute_missing_values.R | tee impute_missing_values.log")
}

grambank = read.table(
  paste0(gb_dir, "/output/GB_wide/GB_wide_imputed_binarized.tsv"),
  sep = "\t",
  header = 1
)
```

Phoible

PHOIBLE is a repository of cross-linguistic phonological inventory data, which have been extracted from source documents and tertiary databases and compiled into a single searchable convenience sample. It contains the cross-linguistic phonological inventory of 2,186 languages, from 176 language families, and a total of 3,183 segment types. For a detailed description of Phoible, see (Moran 2013). The data can be explored at the website: https://phoible.org/

The code below shows how we wrangle to Phoible data into the appropriate format for the multidimensional scaling analysis. Some languages have multiple entries, with varying phonological inventories. We reduce the data to one inventory per language, choosing inventories at random.

```
# phoible data
p_df = read.csv("submodule/phoible_cldf/cldf/values.csv")

# Some languages have been coded multiple times (doculets)
# We select an inventory at random
```

```r
p_ss =  p_df %>%
  dplyr::group_by(Language_ID) %>%
  dplyr::filter(Inventory_ID == sample(unique(Inventory_ID), 1))

# The number of language ids matches the number of Inventory IDs
# (i.e. there is one language code per inventory code)
tt = assert_that(
  n_distinct(p_ss$Language_ID) == n_distinct(p_ss$Inventory_ID)
  )

# Make the dataset wide, this will build a dataset where columns are
# phonemes and rows are languages.
phoible_wide = pivot_wider(p_ss,
                      id_cols = Language_ID,
                      values_from = Value,
                      names_from = Value)

# This changes the data to a 0 (phoneme is not used) 1 (phoneme is used)
phoible_df = apply(phoible_wide[, 2:ncol(phoible_wide)], 2, function(x)
  ifelse(is.na(x), 0, 1))
phoible = data.frame(Language_ID = phoible_wide$Language_ID, phoible_df)
```

The Expanded Graded Intergenerational Disruption Scale (EGIDS)

EGIDS is a 13-point scale of endangerment for 7,206 languages, ranging from 1 (Internationally used languages) to 10 (Language is no longer used, and no one holds a symbolic sense of identity with the language) (Lewis and Simons 2010, Table 1). There are seven categories of 'safe' languages, a vulnerable category, three increasing levels of endangerment, and two categories of languages that are no longer used. Our data collapses the two sleeping categories.

We draw the EGIDS scale from the supplementary data of Bromham et al (2022). We further amend this dataset to a binary category where languages have children speakers or not. Strictly speaking, this is level 7 and above. However, this is a conservative decision. At level 6b, only some children (i.e. not all) are learning the language.

Table 1: Table of the Expanded Graded Intergenerational Disruption Scale, reproduced from Lewis and Simons (2010).

LVL	LABEL	DESCRIPTION	UNESCO

0	International	“The language is used internationally for a broad range of functions.”	Safe
1	National	“The language is used in education, work, mass media, government at the nationwide level.”	Safe
2	Regional	“The language is used for local and regional mass media and governmental services.”	Safe
3	Trade	“The language is used for local and regional work by both insiders and outsiders.”	Safe
4	Educational	“Literacy in the language is being transmitted through a system of public education.”	Safe
5	Written	“The language is used orally by all generations and is effectively used in written form in parts of the community.”	Safe
6a	Vigorous	“The language is used orally by all generations and is being learned by children as their first language.”	Safe

6b	Threatened	"The language is used orally by all generations but only some of the child-bearing generation are transmitting it to their children."	Vulnerable
7	Shifting	"The child-bearing generation knows the language well enough to use it among themselves but non are transmitting it to their children."	Definitely Endangered
8a	Moribund	"The only remaining active speakers of the language are members of the grandparent generation."	Severely Endangered
8b	Nearly Extinct	"The only remaining speakers of the language are members of the grandparent generation or older who have little opportunity to use the language." |	Critically Endangered
9	Dormant	"The language serves as a reminder of heritage identity for an ethnic community. No one has more than symbolic proficiency."	Sleeping

10	Sleeping	"No one retains a sense of ethnic identify associated with the language, even for symbolic purposes."	Sleeping

```
languages = read.csv("processed_data/language_endangerment.csv")

# How many languages are not being taught to children
nochildren_levels = c("6b", "7", "8a", "8b", "9")

## No children learners or not
languages$nochildren = ifelse(languages$EGIDS %in% nochildren_levels,
                              1, 0)
```

Kidd & Garcia (2022)

The supplementary material of Kidd & Garcia have a list of all papers published in the top four child language acquisition journals - including metadata for the language spoken - available here https://osf.io/jmxnw. We have extracted a list of unique languages studied, and the number of papers written about that language into a csv file below. The names used to describe languages have been manually curated to match with the Glottolog dataset. An example of a curated match is ensure languages like *Tongan* in Kidd & Garcia, matches with *Tongan (Tonga Island)* in Glottolog. We require exact matches.

Some languages listed in Kidd & Garcia were not listed in the EGIDS dataset. These languages are listed below.

```
# Raw data from Kidd & Garcia 2022
kiddgarcia = read.csv('data/journal_archive_data_2021.csv', sep = ";")
# Matched data
kiddgarcia_matching = read.csv("processed_data/name_matching.csv")
kiddgarcia = left_join(kiddgarcia, kiddgarcia_matching, by = "language")

# Make sure we count each language once for each paper
## The data is in long format so if a paper is about
## morphosyntax and pragmatics in English,
## English will be listed across two rows.
kiddgarcia_counts = kiddgarcia %>%
  distinct(title, year, volume, issue, journal, egids_matching,
```

```
           .keep_all = TRUE) %>%
  group_by(egids_matching) %>%
  summarise(count = n()) %>%
  na.omit()

languages = left_join(languages, kiddgarcia_counts,
                      by = c("Name" = "egids_matching"))

# Languages that have been studied have a paper count
languages$studied = ifelse(is.na(languages$count), 0, 1)

# Languages that have had a phonological or morphosyntax study about them
kiddgarcia_topics = kiddgarcia %>%
  group_by(language) %>%
  mutate(
    phonology_bin = ifelse(topic == "Phonology", 1, 0),
    morphosyntax_bin = ifelse(topic == "Morphosyntax", 1, 0)
  ) %>%
  summarise(
    phonology_sum = sum(phonology_bin, na.rm = TRUE),
    phonology_studied = phonology_sum > 0,
    morphosyntax_sum = sum(morphosyntax_bin, na.rm = TRUE),
    morphosyntax_studied = morphosyntax_sum > 0
  ) %>%
  na.omit() %>%
  left_join(kiddgarcia_matching, by = "language") %>%
  select(
    egids_matching,
    phonology_sum,
    phonology_studied,
    morphosyntax_sum,
    morphosyntax_studied
  )

languages = left_join(languages, kiddgarcia_topics,
                      by = c("Name" = "egids_matching"))

# find un-matched languages
nm = unique(kiddgarcia$egids_matching) %in% languages$Name
knitr::kable(unique(kiddgarcia$egids_matching)[!nm], col.names = NULL)
```

American Sign Language
Greenlandic
Signing Exact English
Estonian
Home sign
LIS (Italian Sign Language)
Turkish Sign Language

Analysis & Figures

This section contains graphs used in the main text, as well as alternative depictions of the data.

Histogram

This histogram shows the proportion of languages studied by endangerment category, split by whether the languages have been studied or not, according to Kidd & Garcia (2022).

```
global_prop = languages %>%
  group_by(EGIDS.int) %>%
  summarise(n = n(), .groups = 'keep') %>%
  ungroup() %>%
  mutate(freq = n / sum(n)) %>%
  na.omit()

global_prop$studied = 0

studied_prop = languages %>%
  group_by(studied, EGIDS.int) %>%
  summarise(n = n(), .groups = 'keep') %>%
  group_by(studied) %>%
  mutate(freq = n / sum(n)) %>%
  na.omit()

plot_prop = bind_rows(global_prop, studied_prop[studied_prop$studied == 1,])

plot_prop$studied =
  ifelse(plot_prop$studied == 1,
         "Studied Distribution",
```

```
         "Global Distribution")
plot_prop$EGIDS.char = recode(plot_prop$EGIDS.int,
                       `0` = "International",
                       `1` = "National",
                       `2` = "Regional",
                       `3` = "Trade",
                       `4` = "Educational",
                       `5` = "Written",
                       `6` = "Vigorous",
                       `7` = "Threatened",
                       `8` = "Shifting",
                       `9` = "Moribund",
                       `10` = "Nearly Sleeping",
                       `11` = "Sleeping"
                       )

plot_prop$EGIDS.char = factor(plot_prop$EGIDS.char,
                       levels = c("International",
                                  "National",
                                  "Regional",
                                  "Trade",
                                  "Educational",
                                  "Written",
                                  "Vigorous",
                                  "Threatened",
                                  "Shifting",
                                  "Moribund",
                                  "Nearly Sleeping",
                                  "Sleeping"))

# Proportion of unstudied languages that are written, vigorous, or threatened
unstud_prop = studied_prop %>%
  filter(studied == 0 & EGIDS.int >= 5) %>%
  summarise(sum(freq))

p_base = ggplot(plot_prop,
                aes(
                  y = freq,
                  x = EGIDS.char,
                  fill = EGIDS.char,
                  group = studied
                )) +
```

```r
  geom_bar(stat = 'identity') +
  facet_wrap( ~ studied, nrow = 3) +
  ylab("Proportion of languages") +
  xlab("EGIDS") +
  scale_fill_manual(values = c(rep("#009E73", 7),
                               "#E69F00", rep("#D55E00", 4))) +
  theme_classic(base_size = font_size) +
  theme(legend.position = "none",
        axis.text.x = element_text(angle = 45, vjust = 1, hjust = 1))

pp = p_base + geom_bracket(
  xmin = "Moribund",
  xmax = "Sleeping",
  y.position = 0.1,
  tip.length = 0.05,
  vjust = -0.5,
  label = "No children speakers",
  data = data.frame(studied = factor(
    "Global Distribution", levels = c("Studied Distribution",
         "Global Distribution")
  ),
  EGIDS.char = "Sleeping")
)  +
  theme(axis.text.x = element_text(angle = 45, hjust = 1))

ggsave(plot = pp, filename = "figures/figure1.png")

pp
```

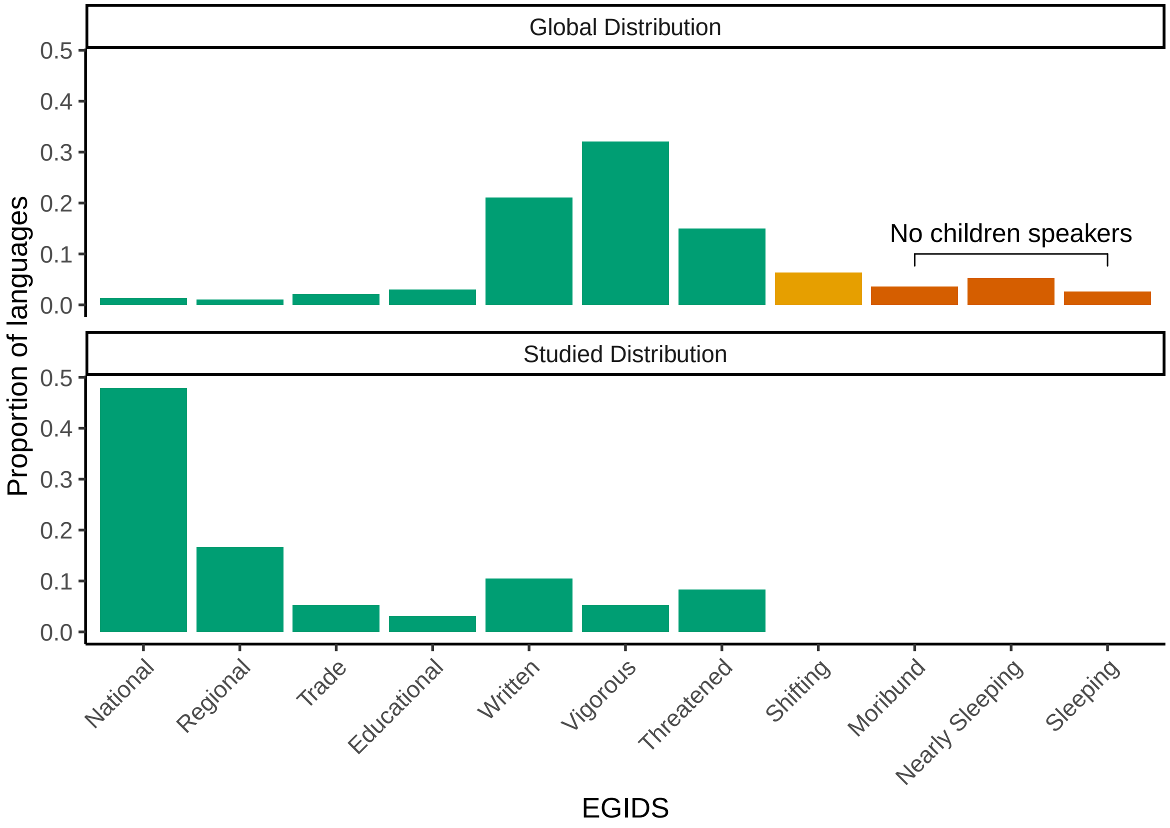

The majority of unstudied languages in the Kidd & Garcia (2022) data set are categorised as written, vigorous, or threatened (87%).

Hexbin graphs

To build the Hexbin projections of linguistic diversity, there are four key steps:

1) Match our dataset of studied languages (Kidd and Garcia 2022), to the Phoible and Grambank datasets

2) Build a distance matrix between all languages

3) Run the multidimensional scaling algorithm

4) Plot and bin the results.

To plot the results, we are required to get custom ggplot code, which has been included in this repository. The custom code was created thanks to Allan Cameron via Stackoverflow.

We do this for the Grambank and Phoible dataset, and for a variety of settings. First, we plot the raw data from the MDS algorithm. Second we plot the data in bins, with no markings.

The next plots highlight bins containing studied languages, first on a raw scale, then on a log scale. We also combine these to create hexbin plot found in the main text. Finally, we create the same plots comparing the spaces studied to unstudied.

At the end of this code block, we show a table containing the languages codes which are dropped from the datasets. Unmatched languages largely occur because Grambank or Phoible identifies a dialect, and Kidd & Garcia contain metadata on languages, or because no data exists in the datasets. In both datasets, dropped languages make up less than 1% of the total sample.

```
# Read in custom Hextri code
source("hextri_grobs.R")

## Build the pipeline as a function
hextri_datacleaning = function(df, languages = languages){
  # ensure the same result everytime
  set.seed(1234)

  # Give data rownames to carry through the analysis
  rownames(df) = df$Language_ID

  # Make distance matrix
  distance_matrix = df %>%
    dplyr::select(-Language_ID) %>%
    cluster::daisy(metric = "gower", warnBin = FALSE)

  # Build Multi-dimensional scaling output
  mds_output = distance_matrix %>%
    cmdscale(eig=TRUE, k=2)

  # Wrangle into a dataframe
  mds_points = data.frame(mds_output$points)
  colnames(mds_points) = c("MDS.X", "MDS.Y")
  mds_points$Glottocode = rownames(mds_points)

  # Join Endangerment and Studied data
  plot_df = left_join(mds_points, languages, by = "Glottocode")
  plot_df = plot_df[, c(
    "Name",
    "Glottocode",
    "MDS.X",
    "MDS.Y",
    "count",
```

```
    "phonology_sum",
    "morphosyntax_sum",
    "nochildren",
    "EGIDS.int"
  )]

  # If there is a missing value,
  # it means no papers were published in the journals reviewed
  plot_df$count[is.na(plot_df$count)] = 0
  plot_df$phonology_sum[is.na(plot_df$phonology_sum)] = 0
  plot_df$morphosyntax_sum[is.na(plot_df$morphosyntax_sum)] = 0

  # Ensure we don't have any missing data otherwise GGplot will complain
  plot_df = plot_df[complete.cases(plot_df),]

  # If a language has at leaast one paper, then it is a studied language
  plot_df$studied = ifelse(plot_df$count > 1, 1, 0)

  # Reverse code the binary variable so that a value of 1 indicates that a
  # language has children speakers
  plot_df$children = 1 - plot_df$nochildren

  # Return plot dataframe
  plot_df
}

# Run pipeline for each dataset
plot_phoible = hextri_datacleaning(phoible, languages = languages)
plot_grambank = hextri_datacleaning(grambank, languages = languages)

# remove outlier
plot_phoible = plot_phoible[plot_phoible$MDS.X > -0.04,]

# Unmatched data
## Phoible
# knitr::kable(phoible$Language_ID[!phoible$Language_ID %in% plot_phoible$Glottocode])
#
# ## Grambank
# knitr::kable(grambank$Language_ID[!grambank$Language_ID %in% plot_grambank$Glottocode])
```

No-bin Plots

The below plots show the output of the multidimensional scaling analysis, without binning.

```
set.seed(1234)
p1_phoible = ggplot(plot_phoible,
                    aes(MDS.X, MDS.Y, col = factor(children))) +
  geom_point(aes(shape = factor(studied), size = phonology_sum + 1),
             alpha = 0.5) +
  coord_equal() +
  theme_classic(base_size = font_size) +
  theme(aspect.ratio = 1, legend.position = "right") +
  scale_linewidth(range = c(0, 3)) +
  scale_alpha(range = c(0.2, 2)) +
  scale_fill_manual(values = c("#D55E00", "#009E73" ),
                    labels = c("No", "Yes")) +
  guides(col=guide_legend(title="Children Speakers"),
         shape=guide_legend(title="Studied")) +
  scale_size(guide = "none") +
  ggtitle("Phoible") +
  xlab("") + ylab("")

p1_grambank = ggplot(plot_grambank,
                     aes(MDS.X, MDS.Y, col = factor(children))) +
  geom_point(aes(shape = factor(studied), size = morphosyntax_sum + 1),
             alpha = 0.5) +
  coord_equal() +
  theme_classic(base_size = font_size) +
  theme(aspect.ratio = 1, legend.position = "right") +
  scale_linewidth(range = c(0, 3)) +
  scale_alpha(range = c(0.2, 2)) +
  scale_fill_manual(values = c("#D55E00", "#009E73"),
                    labels = c("No", "Yes")) +
  guides(col=guide_legend(title="Children Speakers"),
         shape=guide_legend(title="Studied")) +
  scale_size(guide = "none") +
  ggtitle("Grambank") +
  xlab("") + ylab("")

(p1_phoible | p1_grambank) +
  plot_layout(guides = 'collect') &
  theme(legend.position = 'bottom', legend.box="vertical")
```

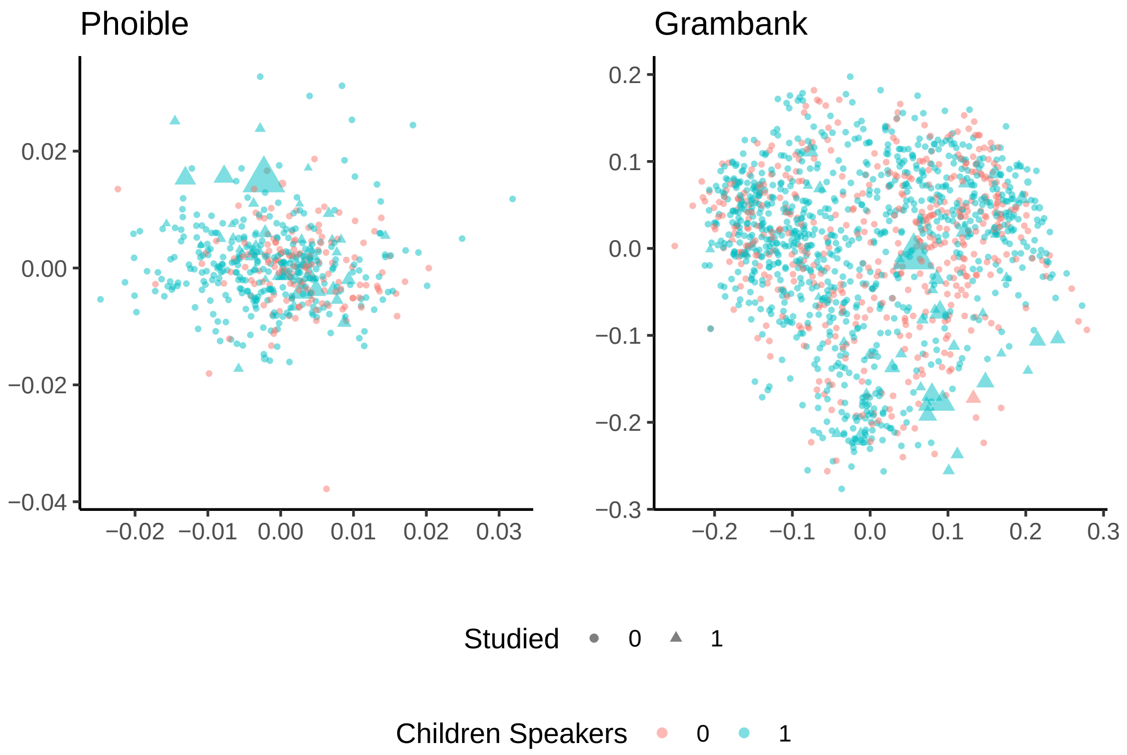

Hexbin Plots

The next section of code shows how we add the hexagonal bins to the plots.

```
n_bins = 14
set.seed(1234)
p2_phoible = ggplot(plot_phoible,
                    aes(MDS.X, MDS.Y, fill = factor(children),
                        col = factor(children))) +
  geom_hextri(color = "white", linewidth = 0.0, bins = n_bins) +
  geom_hex(fill = NA, color = "white", bins = n_bins, lwd = 0.01) +
  coord_equal() +
  theme_classic(base_size = font_size) +
  theme(aspect.ratio = 1, legend.position = "bottom",
        legend.box = "vertical") +
  scale_linewidth(range = c(0, 3)) +
  scale_alpha(range = c(0.2, 2)) +
```

```r
  scale_fill_manual(values = c("#D55E00", "#009E73"),
                    labels = c("No", "Yes")) +
  guides(fill=guide_legend(title="Children Speakers"),
         linewidth=guide_legend(title="Log N Papers"),
         alpha=guide_legend("Count")) +
  ggtitle("Phoible") +
  xlab("") + ylab("")

p2_grambank = ggplot(plot_grambank,
                    aes(MDS.X, MDS.Y, fill = factor(children),
                        col = factor(children))) +
  geom_hextri(color = "white", linewidth = 0.01, bins = n_bins) +
  geom_hex(fill = NA, color = "white", bins = n_bins, lwd = 0.01) +
  coord_equal() +
  theme_classic(base_size = font_size) +
  theme(aspect.ratio = 1, legend.position = "bottom", legend.box="vertical") +
  scale_linewidth(range = c(0, 3)) +
  scale_alpha(range = c(0.2, 2)) +
  scale_fill_manual(values = c("#D55E00", "#009E73"),
                    labels = c("No", "Yes")) +
  guides(fill=guide_legend(title="Children Speakers"),
         linewidth=guide_legend(title="Log N Papers"),
         alpha = guide_legend(title = "Count")) +
  ggtitle("Grambank") +
  xlab("") + ylab("")

p2_phoible
```

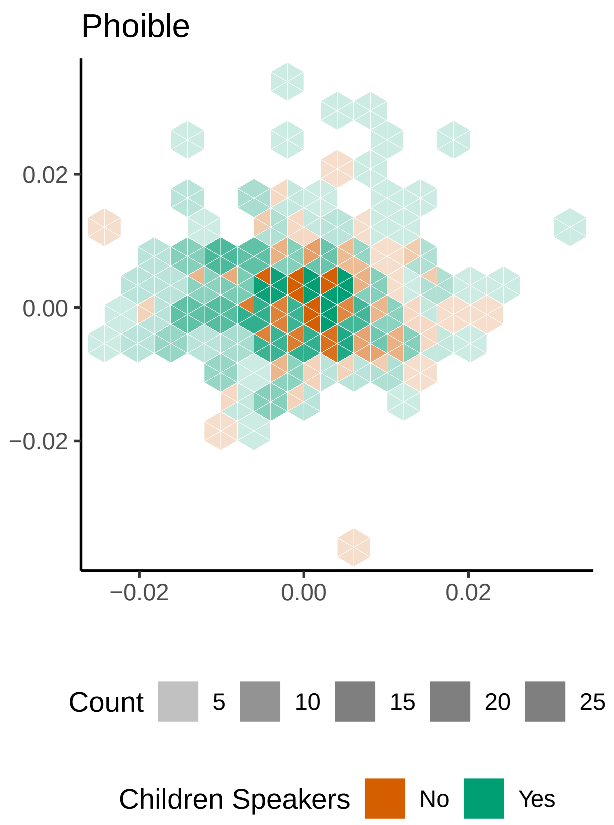

Phoible
0.02
0.00
−0.02
−0.02
0.00
0.02
Count
5
10
15
20
25
Children Speakers
No
Yes


```
p2_grambank
```

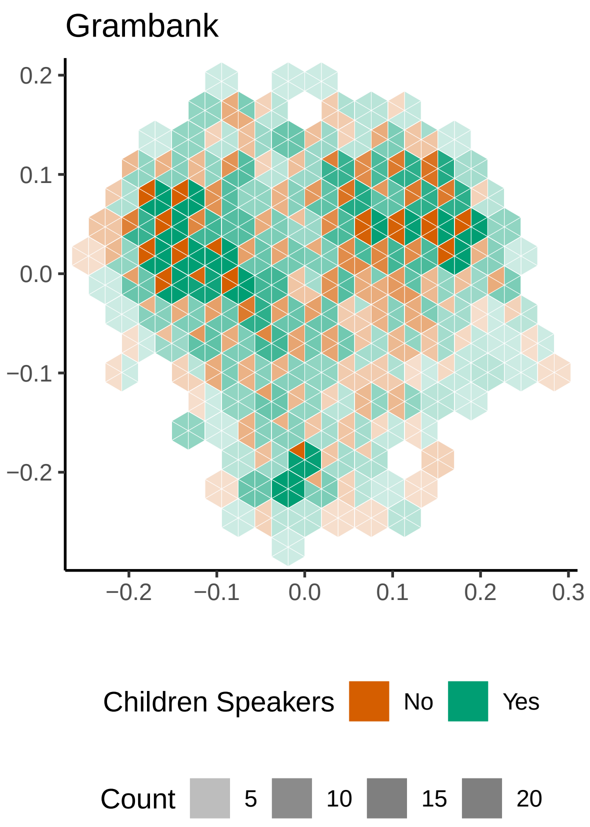

Hexbin & Studied Languages Plots

The next code section shows how we highlight cells that have been studied, and to weight those cells by the number of papers published.

```
set.seed(1234)
p3_phoible = ggplot(plot_phoible,
                    aes(MDS.X, MDS.Y, fill = factor(children),
                        col = factor(children))) +
  geom_hextri(color = "white", linewidth = 0.0, bins = 15) +
  geom_hex(fill = NA, color = "white", bins = 15, lwd = 0.05) +
  stat_summary_hex(aes(
    z = phonology_sum,
    linewidth = after_stat(value),
  ), fun = ~ sum((.x[.x > 0])), col = "black", fill = NA, bins = 15) +
  coord_equal() +
  theme_classic(base_size = font_size) +
```

```r
  theme(aspect.ratio = 1, legend.position = "right") +
  scale_linewidth(range = c(0, 3)) +
  scale_alpha(range = c(0.2, 2)) +
  scale_fill_manual(values = c("#D55E00", "#009E73"),
                    labels = c("No", "Yes")) +
  guides(fill=guide_legend(title="Children Speakers"),
         linewidth=guide_legend(title="N Papers"),
         alpha=guide_legend(title="N Languages")) +
  ggtitle("Phoible") +
  xlab("") + ylab("")

p3_grambank = ggplot(plot_grambank,
                    aes(MDS.X, MDS.Y, fill = factor(children),
                        col = factor(children))) +
  geom_hextri(color = "white", linewidth = 0.0, bins = 15) +
  geom_hex(fill = NA, color = "white", bins = 15, lwd = 0.05) +
  stat_summary_hex(aes(
    z = morphosyntax_sum,
    linewidth = after_stat(value),
  ), fun = ~ sum((.x[.x > 0])), col = "black", fill = NA, bins = 15) +
  coord_equal() +
  theme_classic(base_size = font_size) +
  theme(aspect.ratio = 1, legend.position = "right") +
  scale_linewidth(range = c(0, 3),
                  breaks = c(0, 250, 500, 1000)) +
  scale_alpha(range = c(0.2, 2)) +
  scale_fill_manual(values = c("#D55E00", "#009E73"),
                    labels = c("No", "Yes")) +
  guides(fill=guide_legend(title="Children Speakers"),
         linewidth=guide_legend(title="N Papers"),
         alpha=guide_legend(title="N Languages")) +
  ggtitle("Grambank") +
  xlab("") + ylab("")

p3_phoible
```

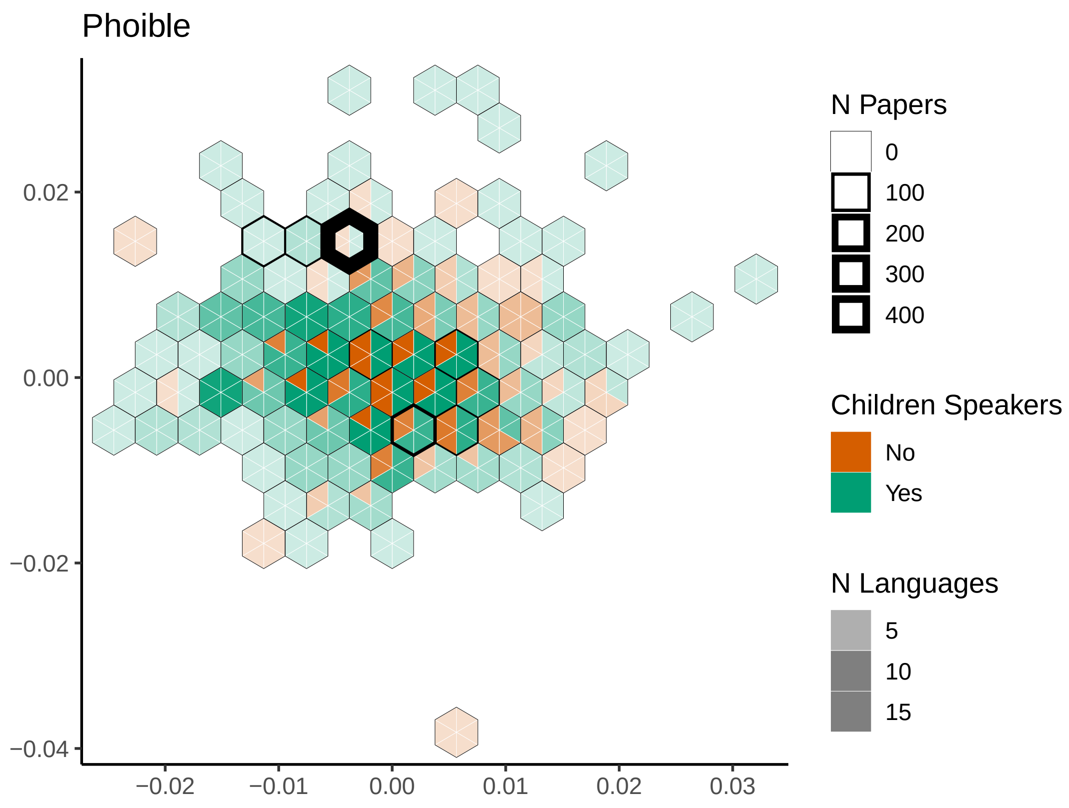
Phoible
0.02
0.00
−0.02
−0.04
−0.02
−0.01
0.00
0.01
0.02
0.03
N Papers
0
100
200
300
400
Children Speakers
No
Yes
N Languages
5
10
15

```
p3_grambank
```

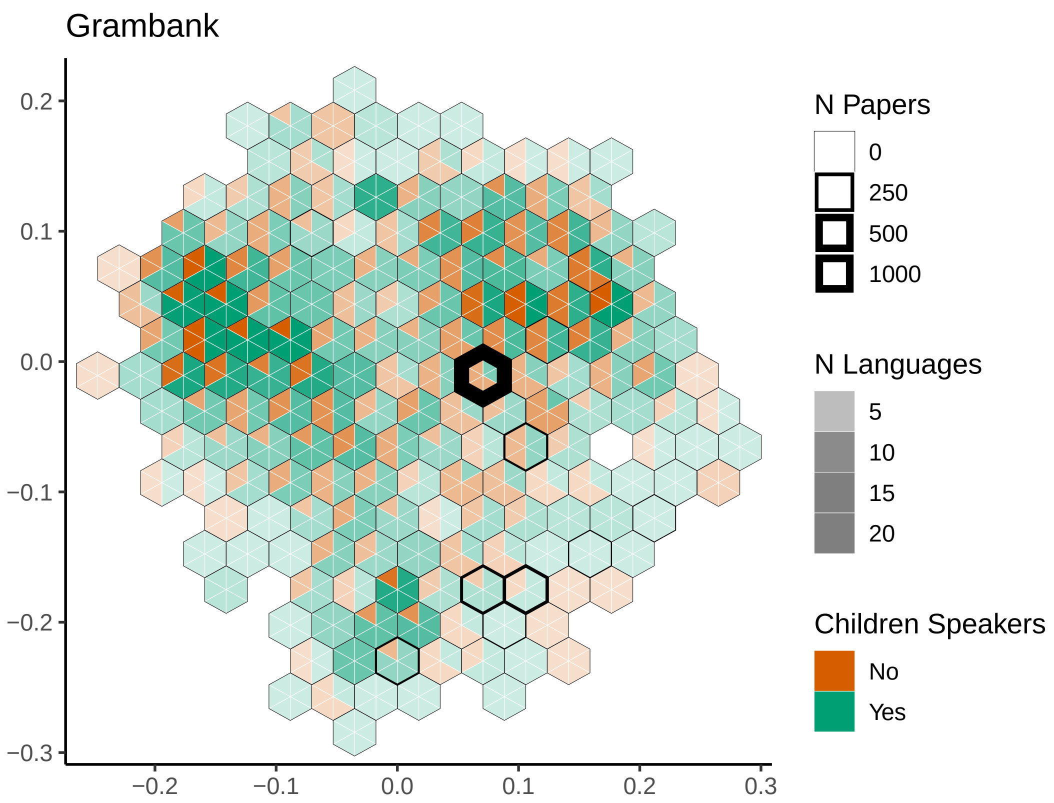

Hexbin & Studied Languages (Log Scale) Plots

The next set of code shows how we re-scale the weightings of papers published onto the log-scale.

```
set.seed(1234)
p4_phoible = ggplot(plot_phoible,
                    aes(MDS.X, MDS.Y, fill = factor(children),
                        col = factor(children))) +
  geom_hextri(color = "white", linewidth = 0.0, bins = 15) +
  geom_hex(fill = NA, color = "white", bins = 15, lwd = 0.05) +
  stat_summary_hex(aes(
    z = count,
    linewidth = after_stat(value),
  ), fun = ~ sum((.x[.x > 0])), col = "black", fill = NA, bins = 15) +
  coord_equal() +
  theme_classic(base_size = font_size) +
```

```
  theme(aspect.ratio = 1, legend.position = "right") +
  scale_linewidth(range = c(0, 3),
                  trans = scales::pseudo_log_trans(base = 10)) +
  scale_alpha(range = c(0.2, 2)) +
  scale_fill_manual(values = c("#D55E00", "#009E73"),
                    labels = c("No", "Yes")) +
  guides(fill=guide_legend(title="Children Speakers"),
         linewidth=guide_legend(title="Log N Papers"),
         alpha=guide_legend(title="N Languages")) +
  ggtitle("Phoible") +
  xlab("") + ylab("")

p4_grambank = ggplot(plot_grambank,
                    aes(MDS.X, MDS.Y, fill = factor(children),
                        col = factor(children))) +
  geom_hextri(color = "white", linewidth = 0.0, bins = 15) +
  geom_hex(fill = NA, color = "white", bins = 15, lwd = 0.05) +
  stat_summary_hex(aes(
    z = count,
    linewidth = after_stat(value),
  ), fun = ~ sum(.x[.x > 0]), col = "black", fill = NA, bins = 15) +
  coord_equal() +
  theme_classic(base_size = font_size) +
  theme(aspect.ratio = 1, legend.position = "right") +
  scale_linewidth(range = c(0, 3),
                  trans = scales::pseudo_log_trans(base = 10),
                  breaks = c(0, 10, 100, 1000)) +
  scale_alpha(range = c(0.2, 2)) +
  scale_fill_manual(values = c("#D55E00", "#009E73"),
                    labels = c("No", "Yes")) +
  guides(fill=guide_legend(title="Children Speakers"),
         linewidth=guide_legend(title="Log N Papers"),
         alpha=guide_legend(title="N Languages")) +
  ggtitle("Grambank") +
  xlab("") + ylab("")

p4_phoible
```

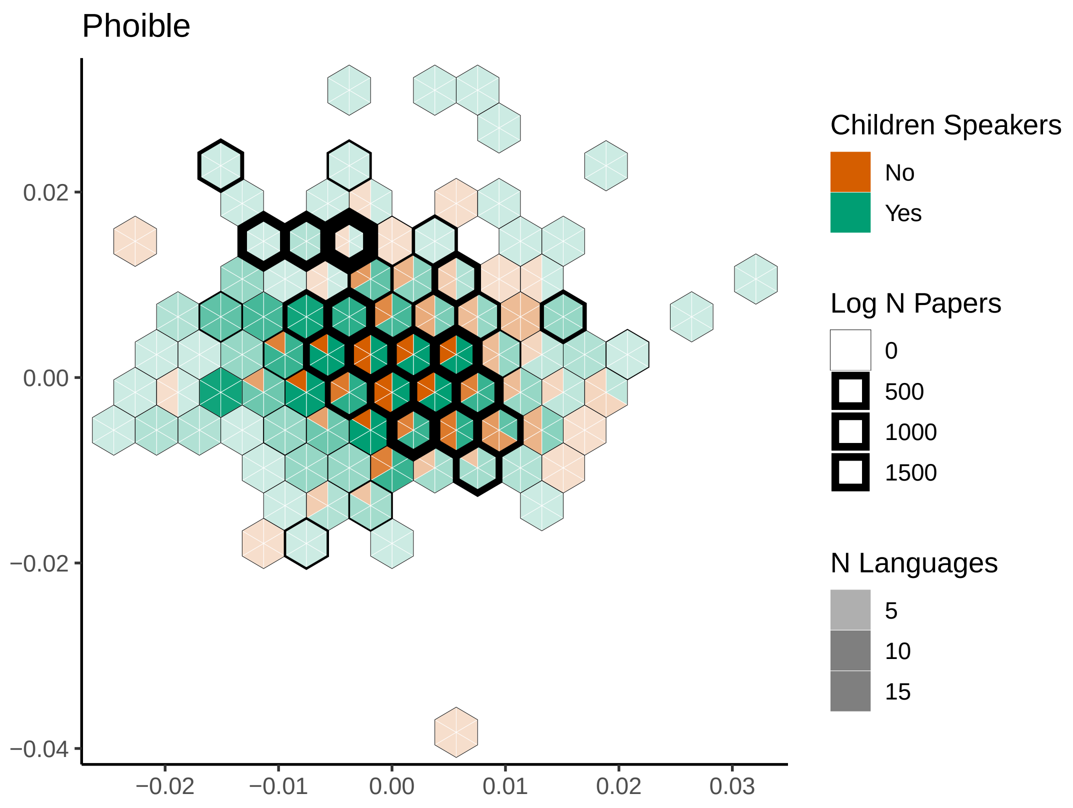
Phoible
0.02
0.00
−0.02
−0.04
−0.02
−0.01
0.00
0.01
0.02
0.03
Children Speakers
No
Yes
Log N Papers
0
500
1000
1500
N Languages
5
10
15

```
p4_grambank
```

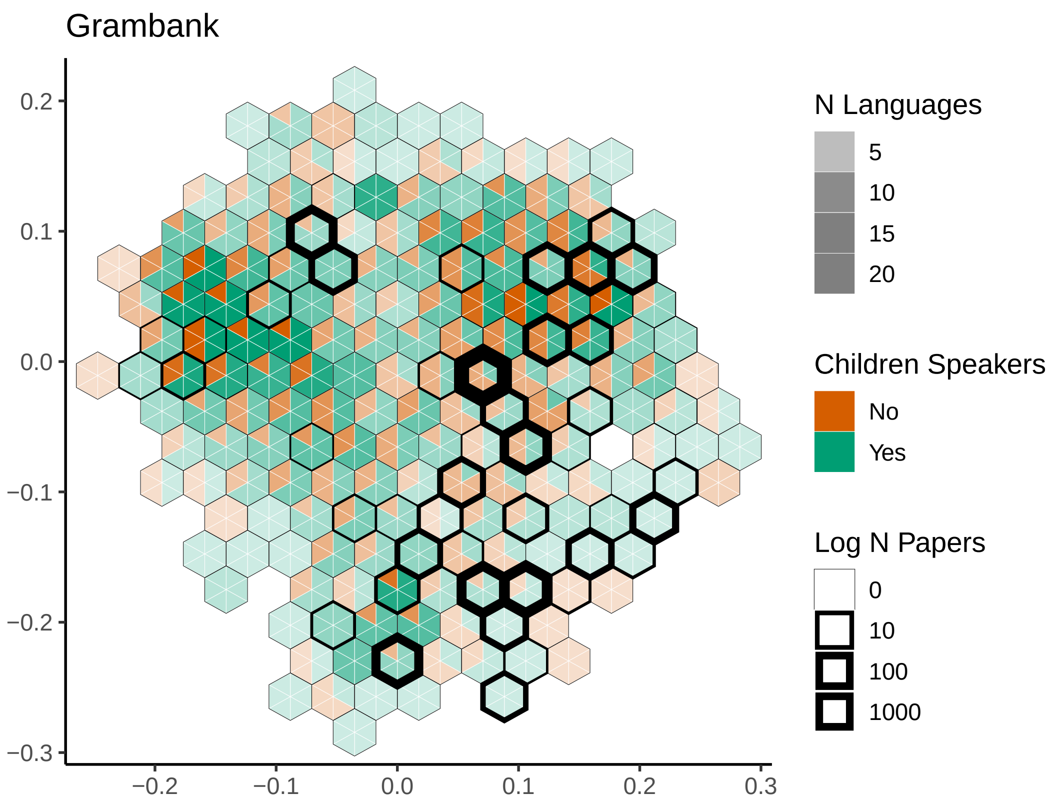

Summary Statistics

The next chunk of code will extract the summary information from the plots, for discussion in the text.

```
### Count data ###
papercount_pb = layer_data(p3_phoible, 3)
papercount_gb = layer_data(p3_grambank, 3)

# how many cells are there
pb.s = length(papercount_pb$value)
gb.s = length(papercount_gb$value)

cat("In Phoible plots there are", pb.s, "cells.\n")
```

```
In Phoible plots there are 92 cells.
```

```
cat("In Grambank plots there are", gb.s, "cells.\n")
```

```
In Grambank plots there are 184 cells.
```

```
# how many cells have been studied
pb.cs = sum(papercount_pb$value != 0)
gb.cs = sum(papercount_gb$value != 0)

cat("In Phoible plots,", pb.cs / pb.s, "cells have been studied")
```

```
In Phoible plots, 0.3152174 cells have been studied
```

```
cat("In Phoible plots,", gb.cs / gb.s, "cells have been studied")
```

```
In Phoible plots, 0.2663043 cells have been studied
```

```
# n papers in one cell
cat("In the Phoible plot",
    round(max(papercount_pb$value) / sum(papercount_pb$value), 2),
    "of papers are in the most populated cell.\n")
```

```
In the Phoible plot 0.47 of papers are in the most populated cell.
```

```
cat("In the Grambank plot,",
    round(max(papercount_gb$value) / sum(papercount_gb$value), 2),
    "of papers are in the most populated cell.\n")
```

```
In the Grambank plot, 0.4 of papers are in the most populated cell.
```

```
# how many studied cells have less than 10 papers
pb.10 = sum(papercount_pb$value <= 10 & papercount_pb$value != 0)
gb.10 = sum(papercount_gb$value <= 10 & papercount_gb$value != 0)

pb.10p = pb.10 / sum(papercount_pb$value != 0)
gb.10p = gb.10 / sum(papercount_gb$value != 0)

cat("There are", pb.10, "cells in Phoible that have less than 10 papers (",
    scales::percent(pb.10 / pb.cs), ").\n From a total of ,", pb.cs,
```

```
    " studied cells\n",
    "and there are", gb.10,
    "cells in Grambank (", scales::percent(gb.10 / gb.cs),
    ").\nFrom a total of", gb.cs, "studied cells.\n")
```

```
There are 16 cells in Phoible that have less than 10 papers ( 55% ).
 From a total of , 29  studied cells
 and there are 27 cells in Grambank ( 55% ).
From a total of 49 studied cells.
```

```
# extract data from ggplot
pb_pdata.1 = layer_data(p3_phoible, 1)
gb_pdata.1 = layer_data(p4_grambank, 1)

pb_pdata.2 = layer_data(p3_phoible, 2)
gb_pdata.2 = layer_data(p4_grambank, 2)

pb_pdata.3 = layer_data(p3_phoible, 3)
gb_pdata.3 = layer_data(p4_grambank, 3)

# Hexbins counts
# number of bins
n_bins.pb = nrow(pb_pdata.2)
n_bins.gb = nrow(gb_pdata.2)

# number of studied bins
s_bins.pb = sum(pb_pdata.3$value > 0)
s_bins.gb = sum(gb_pdata.3$value > 0)

s_bins.pbp = s_bins.pb / nrow(pb_pdata.2)
s_bins.gbp = s_bins.gb / nrow(gb_pdata.2)

cat("There are", n_bins.pb, "in the Phoible plot.",
    s_bins.pb, "have been studied (", scales::percent(s_bins.pbp), ").")
```

```
There are 92 in the Phoible plot. 29 have been studied ( 32% ).
```

```
cat("There are", n_bins.gb, "in the Grambank plot.",
    s_bins.gb, "have been studied (", scales::percent(s_bins.gbp), ").")
```

```
There are 184 in the Grambank plot. 53 have been studied ( 29% ).
```

Hexbin Variation tests

Our Hexbin statistics are based on the number of languages within each hexbin in the graph. Size of hexbins are a parameter chosen by us, however. Here, we show that increasing the size of hexbins only affects the counts but not proportion of cells that are studied or unstudied, with these variables showing a strong positive correlation.

```
n_bins.pb = c()
n_bins.gb = c()
s_bins.pb = c()
s_bins.gb = c()

bin_size = seq(from = 5, to = 40, by = 5)

for(i in seq_along(bin_size)){
  plot_phoible = hextri_datacleaning(phoible, languages = languages)
  plot_grambank = hextri_datacleaning(grambank, languages = languages)

  p3_phoible = ggplot(plot_phoible,
                  aes(MDS.X, MDS.Y, fill = factor(children),
                      col = factor(children))) +
  geom_hextri(color = "white", linewidth = 0.0, bins = bin_size[i]) +
  geom_hex(fill = NA, color = "white", bins = bin_size[i], lwd = 0.05) +
  stat_summary_hex(aes(
    z = phonology_sum,
    linewidth = after_stat(value),
  ), fun = ~ sum((.x[.x > 0])), col = "black", fill = NA, bins = bin_size[i]) +
  coord_equal() +
  theme_classic(base_size = font_size) +
  theme(aspect.ratio = 1, legend.position = "right") +
  scale_linewidth(range = c(0, 3)) +
  scale_alpha(range = c(0.2, 2)) +
  scale_fill_manual(values = c("#D55E00", "#009E73"),
                  labels = c("No", "Yes")) +
  guides(fill=guide_legend(title="Children Speakers"),
         linewidth=guide_legend(title="N Papers"),
         alpha=guide_legend(title="N Languages")) +
  ggtitle("Phoible") +
  xlab("") + ylab("")

  p3_grambank = ggplot(plot_grambank,
                  aes(MDS.X, MDS.Y, fill = factor(children),
                      col = factor(children))) +
```

```
  geom_hextri(color = "white", linewidth = 0.0, bins = bin_size[i]) +
  geom_hex(fill = NA, color = "white", bins = bin_size[i], lwd = 0.05) +
  stat_summary_hex(aes(
    z = morphosyntax_sum,
    linewidth = after_stat(value),
  ), fun = ~ sum((.x[.x > 0])), col = "black", fill = NA, bins = bin_size[i]) +
  coord_equal() +
  theme_classic(base_size = font_size) +
  theme(aspect.ratio = 1, legend.position = "right") +
  scale_linewidth(range = c(0, 3),
                  breaks = c(0, 250, 500, 1000)) +
  scale_alpha(range = c(0.2, 2)) +
  scale_fill_manual(values = c("#D55E00", "#009E73"),
                    labels = c("No", "Yes")) +
  guides(fill=guide_legend(title="Children Speakers"),
         linewidth=guide_legend(title="N Papers"),
         alpha=guide_legend(title="N Languages")) +
  ggtitle("Grambank") +
  xlab("") + ylab("")

  pb_pdata.2 = layer_data(p3_phoible, 2)
  gb_pdata.2 = layer_data(p3_grambank, 2)

  pb_pdata.3 = layer_data(p3_phoible, 3)
  gb_pdata.3 = layer_data(p3_grambank, 3)

  # Hexbins counts
  # number of bins
  n_bins.pb[i] = nrow(pb_pdata.2)
  n_bins.gb[i] = nrow(gb_pdata.2)

  # number of studied bins
  s_bins.pb[i] = sum(pb_pdata.3$value > 0)
  s_bins.gb[i] = sum(gb_pdata.3$value > 0)
}

var_pb = data.frame(n_bins = n_bins.pb, studied_bins = s_bins.pb,
                    dataset = "Phoible", label = bin_size)
var_gb = data.frame(n_bins = n_bins.gb, studied_bins = s_bins.gb,
                    dataset = "Grambank", label = bin_size)
var_df = rbind(var_pb, var_gb)
```

```r
ggplot(var_df, aes(x = n_bins, y = studied_bins,
                   col = dataset, label = label)) +
  geom_text() +
  ylab("Number of studied bins") +
  xlab("Total number of bins")
```

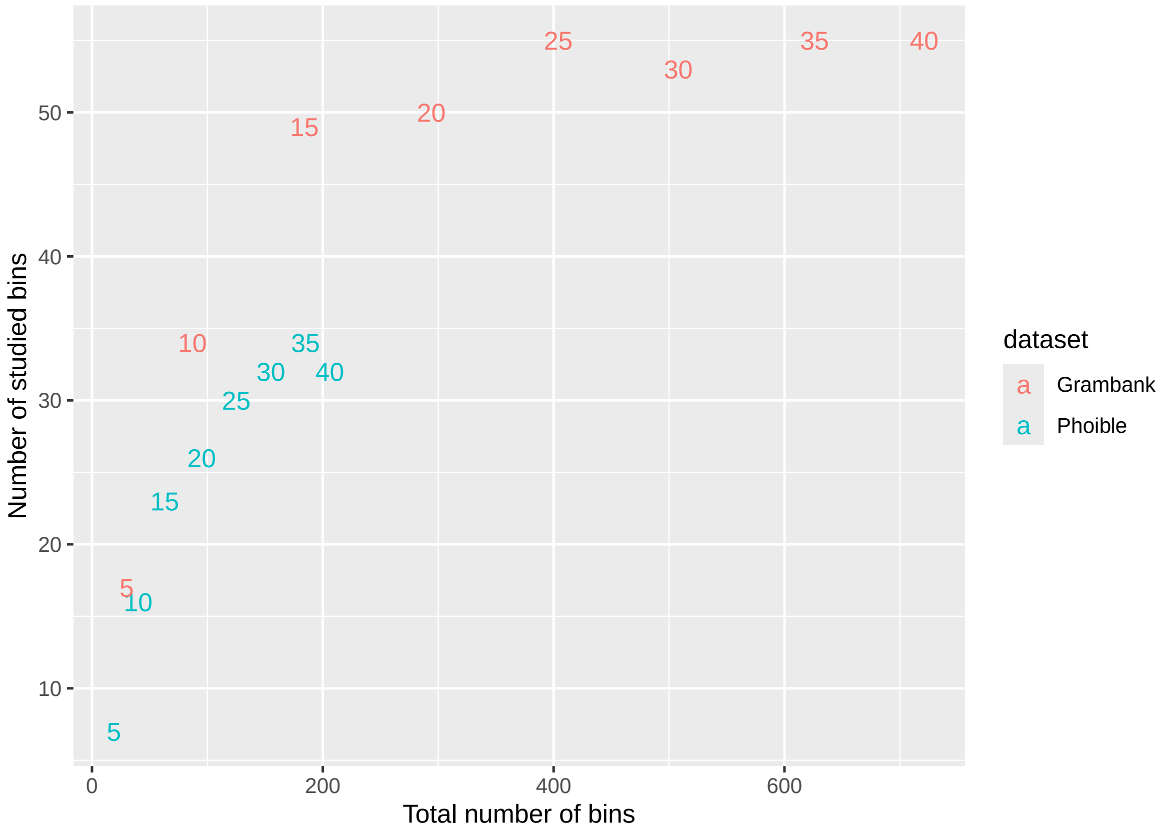

Language Endangerment & ARIMA models

Here, we detail the langauge endangerment data, and ARIMA modelling.

First, we show how many languages are shifting between different EGIDS endangerment categories, based on the supplementary material from Bromham et al. (2022).

```r
bromham_probabilities = readxl::read_xlsx("data/Bromham_suppdata.xlsx",
                                          sheet = 4,
                                          skip = 2)
colnames(bromham_probabilities)[1] = "ISO"
```

```
bromham_2060 = bromham_probabilities %>%
  select(ISO, contains("40"))

bromham_2060$EGIDS.value =
  apply(bromham_2060[, 2:ncol(bromham_2060)], 1, function(x)
    names(x)[x == max(x)])
bromham_2060$EGIDS.value = case_match(
  bromham_2060$EGIDS.value,
  "P[Y=1].40" ~ 1,
  "P[Y=2].40" ~ 2,
  "P[Y=5].40" ~ 5,
  "P[Y=7].40" ~ 7
)

# Current EGIDS score vs Predicted EGIDS score in 40 years time
bromham_nowvs40y = data.frame(
  now = bromham_probabilities$`1=1-6a, 2=6b, 3=7, 4=8a, 5=8b, 6=9, 7=10`,
  future = bromham_2060$EGIDS.value
)

bromham_summary = bromham_2060 %>%
  group_by(EGIDS.value) %>%
  summarise(n = n()) %>%
  mutate(freq = n / sum(n))

bromham_summary = data.frame(studied = "2060",
                             EGIDS = bromham_summary$EGIDS.value,
                             n = bromham_summary$n,
                             freq = bromham_summary$freq)
```

To show how many new languages are being studied, and how that might be projected into the future, we use a simple ARIMA model of the number of new languages studied each year, predicted by the number of papers published per year. We calculate how many more papers are being published per year.

```
# What languages are studied in what year
years = split(kiddgarcia$language, f = kiddgarcia$year)

# Determine which years new languages were studied
by_year = data.frame(year = names(years))
by_year$total_languages = NA
```

```r
# How many languages were studied in year 1
# which languages
studied_languages = unique(years[[1]])
# number of unique languages
by_year$total_languages[1] = n_distinct(studied_languages)

for(i in 2:length(years)){
  # what languages were studied this year
  new_year = unique(years[[i]])

  # How many of those are new languages
  new_total = by_year$total_languages[i - 1] +
    sum(!new_year %in% studied_languages)
  by_year$total_languages[i] = new_total

  # add the new languages to the studied languages pile
  new_languages = new_year[!new_year %in% studied_languages]
  studied_languages = c(studied_languages, new_languages)
}

# Calculate the number of new languages studied within each year
by_year$new_langs = c(7, diff(by_year$total_languages))

# calculate the number of papers studied in each year
papers_byyear = kiddgarcia %>%
  distinct(title, issue, volume, year) %>%
  group_by(year) %>%
  summarise(n_articles = n()) %>%
  mutate(year = as.character(year))

by_year = left_join(by_year, papers_byyear, by = "year")

# check final value is right
tt = assert_that(by_year$total_languages[nrow(by_year)] ==
                   n_distinct(kiddgarcia$language))

## Paper summary over time
# Papers per year between 1980 and 2000
by_year %>% filter(year >= 1980 &
                     year <= 2000) %>%
  pull(n_articles) %>%
  summary()
```

```
   Min. 1st Qu.  Median    Mean 3rd Qu.    Max.
  40.00   47.00   51.00   51.29   55.00   65.00
```

```r
# Papers per year between 2001 and 2020
by_year %>% filter(year >= 2001 &
                     year <= 2021) %>%
  pull(n_articles) %>%
  summary()
```

```
   Min. 1st Qu.  Median    Mean 3rd Qu.    Max.
   52.0    76.5    90.0    93.8   112.0   138.0
```

```r
#### Model ####
ts_data = ts(by_year, start = c(1974, 1), frequency = 1)

ARIMAfit = forecast::tslm(new_langs ~ n_articles, data = ts_data)
summary(ARIMAfit)
```

```

Call:
forecast::tslm(formula = new_langs ~ n_articles, data = ts_data)

Residuals:
    Min      1Q  Median      3Q     Max
-2.2096 -1.1463 -0.1181  0.7763  5.1352

Coefficients:
            Estimate Std. Error t value Pr(>|t|)
(Intercept) 1.745211   0.607632   2.872   0.0062 **
n_articles  0.007037   0.008299   0.848   0.4010
---
Signif. codes:  0 '***' 0.001 '**' 0.01 '*' 0.05 '.' 0.1 ' ' 1

Residual standard error: 1.749 on 45 degrees of freedom
Multiple R-squared:  0.01572,   Adjusted R-squared:  -0.00615
F-statistic: 0.7188 on 1 and 45 DF,  p-value: 0.401
```

The ARIMA model shows that there is no significant relationship between the increase in the number of papers being published and the number of new languages being studied.

Plot ARIMA

```
future_forcasts =
  forecast(ARIMAfit,
           newdata = data.frame(
             year = 2021:2060,
             n_articles = rnorm(40, mean = 130, sd = 5)
           ))

plot_arima = data.frame(
  year = as.numeric(by_year$year),
  newlanguages_year = by_year$new_langs,
  newpapers_year = by_year$n_articles
)

plot_arima$cumsum_languages = cumsum(plot_arima$newlanguages_year)

plot_arima_pred = data.frame(
  year = c(future_forcasts$newdata$year),
  newlanguages_year = c(future_forcasts$mean),
  newlanguages_yearL = c(future_forcasts$lower[,1]),
  newlanguages_yearU = c(future_forcasts$upper[,1])
)

base_2020 = tail(plot_arima$cumsum_languages, 1)

plot_arima_pred$cumsum_languages =
  cumsum(plot_arima_pred$newlanguages_year) + base_2020
plot_arima_pred$cumsum_languagesL =
  cumsum(plot_arima_pred$newlanguages_yearL) + base_2020
plot_arima_pred$cumsum_languagesU =
  cumsum(plot_arima_pred$newlanguages_yearU) + base_2020

## Accounting for expected languages loss
# 72 languages a year shifting to no children speakers
cumulativeloss_children = cumsum(rep(72, nrow(plot_arima_pred) - 1))
starting_point = tail(plot_arima$cumsum_languages, 1)
plot_arima_pred$cumsum_languages_nochildren =
  c(starting_point,
    plot_arima_pred$cumsum_languages[2:nrow(plot_arima_pred)] -
      cumulativeloss_children)
```

```r
plot_arima_pred$cumsum_languages_nochildrenU =
  c(starting_point,
    plot_arima_pred$cumsum_languagesU[2:nrow(plot_arima_pred)] -
      cumulativeloss_children)

plot_arima_pred$cumsum_languages_nochildrenL =
  c(starting_point,
    plot_arima_pred$cumsum_languagesL[2:nrow(plot_arima_pred)] -
      cumulativeloss_children)

# ggplot wont draw any error lines if they fall outside the plotting area
# So we alter the last values to the edge of the plotting zone
plot_arima_pred$cumsum_languages_nochildren[3] = 5
plot_arima_pred$cumsum_languages_nochildrenL[3] = 0.1
plot_arima_pred$cumsum_languages_nochildrenU[3] = 10

# 12 languages a year becoming endangered
cumulativeloss_endangered = cumsum(rep(12, nrow(plot_arima_pred) - 1))

plot_arima_pred$cumsum_languages_endangered =
  c(starting_point,
    plot_arima_pred$cumsum_languages[2:nrow(plot_arima_pred)] -
      cumulativeloss_endangered)

plot_arima_pred$cumsum_languages_endangeredU = c(starting_point,
    plot_arima_pred$cumsum_languagesU[2:nrow(plot_arima_pred)] -
      cumulativeloss_endangered)

plot_arima_pred$cumsum_languages_endangeredL =
  c(starting_point,
    plot_arima_pred$cumsum_languagesL[2:nrow(plot_arima_pred)] -
      cumulativeloss_endangered)

# ggplot wont draw any error lines if they fall outside the plotting area
# So we alter the last values to the edge of the plotting zone

plot_arima_pred$cumsum_languages_endangeredL[10:16] = 0.1
plot_arima_pred$cumsum_languages_endangered[12:16] = 0.1

# No NAs to avoid GGplot messages
plot_arima_pred[plot_arima_pred < 0] = NA
```

```r
pp.1 =
  ggplot(data = plot_arima,
         aes(x = year, y = cumsum_languages, group = 1)) +
  geom_line() +
  # Predicted increase
  geom_smooth(data = plot_arima_pred,
              aes(y = cumsum_languages, x = year,
                  ymin = cumsum_languagesL, ymax = cumsum_languagesU),
              col = "grey", stat = "identity", alpha = 0.5) +
  theme_classic(base_size = font_size) +
  # Children spoken languages losees
  geom_line(data = plot_arima_pred,
            aes(x = year, y = cumsum_languages_nochildren),
            col = "#D55E00", lty = "dashed") +
  geom_smooth(data = plot_arima_pred,
              aes(x = year, y = cumsum_languages_nochildren,
                  ymin = cumsum_languages_nochildrenL,
                  ymax = cumsum_languages_nochildrenU),
              fill = "#D55E00", stat = "identity", linetype=0) +
  # Spoken Language plot_arima_pred
  geom_line(data = plot_arima_pred,
            aes(x = year, y = cumsum_languages_endangered),
            col = "#f2ac42", lty = "dotted") +
  geom_smooth(data = plot_arima_pred,
              aes(x = year, y = cumsum_languages_endangered,
                  ymin = cumsum_languages_endangeredL,
                  ymax =cumsum_languages_endangeredU),
              fill = "#f2ac42", stat = "identity", linetype=0) +
  xlab("Years") +
  ylab("N languages studied") +
  geom_vline(aes(xintercept = 2020.5), col = "grey", lty = "dashed") +
  ggtitle("Predicted Increase in number of languages studied",
          subtitle = "Against predicted rate of language loss")

## new languages per year against papers
plot_arima$cumsum_papers = cumsum(plot_arima$newpapers_year)

pp.2 =
  ggplot(data = plot_arima,
         aes(x = year, y = newlanguages_year, group = 1)) +
  geom_line() +
  # Number of papers per year
```

```
  geom_col(aes(x = year, y = newpapers_year), alpha = 0.4) +
  theme_classic(base_size = font_size) +
  xlab("Years") +
  ylab("Count") +
  ggtitle("Counts of New Languages (line) and Number of Papers (bars)")


stacked =  pp.2 / pp.1
stacked
```

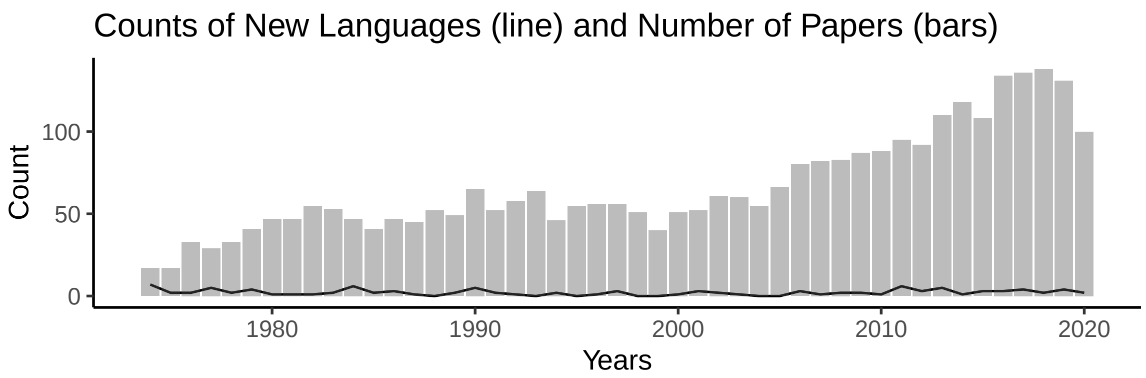

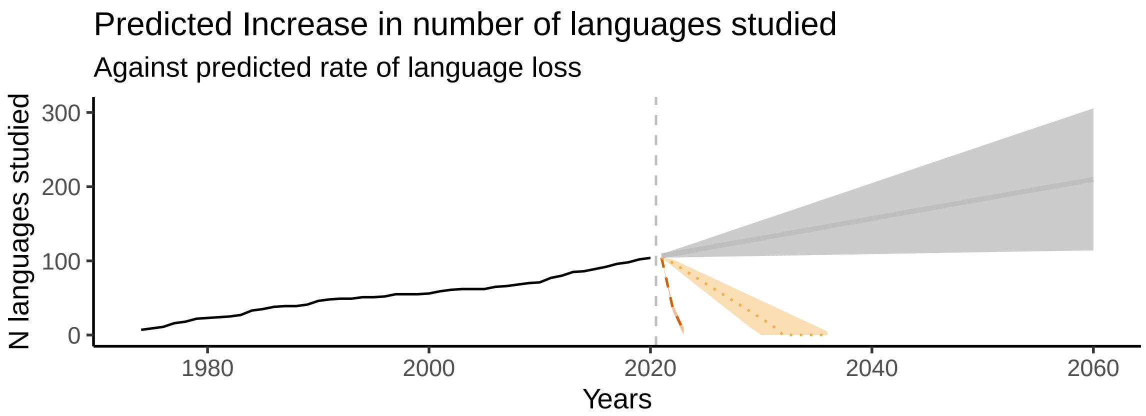

References

Bromham, Lindell, Russell Dinnage, Hedvig Skirgård, Andrew Ritchie, Marcel Cardillo, Felicity Meakins, Simon Greenhill, and Xia Hua. 2021. "Global Predictors of Language Endangerment and the Future of Linguistic Diversity." *Nature Ecology & Evolution* 6 (2): 163–73. https://doi.org/10.1038/s41559-021-01604-y.

Kidd, Evan, and Rowena Garcia. 2022. "How Diverse Is Child Language Acquisition Research?" *First Language* 42 (6): 703–35. https://doi.org/10.1177/01427237211066405.

Lewis, M. Paul, and Gary F. Simons. 2010. "Assessing Endangerment: Expanding Fishman's GIDS." *Revue Roumaine de Linguistique*, no. 2: 103–20.

Moran, Steven. 2013. "Phonetics Information Base and Lexicon." PhD thesis, Max Planck Institute for the Science of Human History. https://doi.org/https://doi.org/10.5281/zenodo.2593234.

Moran, Steven, and Daniel McCloy. 2019. "PHOIBLE 2.0." https://doi.org/10.5281/zenodo.2593234.

Skirgård, Hedvig, Hannah J. Haynie, Damián E. Blasi, Harald Hammarström, Jeremy Collins, Jay J. Latarche, Jakob Lesage, et al. 2023. "Grambank Reveals the Importance of Genealogical Constraints on Linguistic Diversity and Highlights the Impact of Language Loss." *Science Advances* 9 (16): eadg6175. https://doi.org/10.1126/sciadv.adg6175.
